# CD116^+^ fetal precursors migrate to the perinatal lung and give rise to human alveolar macrophages

**DOI:** 10.1084/jem.20210987

**Published:** 2022-01-12

**Authors:** Elza Evren, Emma Ringqvist, Jean-Marc Doisne, Anna Thaller, Natalie Sleiers, Richard A. Flavell, James P. Di Santo, Tim Willinger

**Affiliations:** 1 Center for Infectious Medicine, Department of Medicine Huddinge, Karolinska Institutet, Karolinska University Hospital, Stockholm, Sweden; 2 Innate Immunity Unit, Institut Pasteur, Paris, France; 3 Institut national de la santé et de la recherche médicale U1223, Paris, France; 4 Université de Paris, Sorbonne Paris Cité, Paris, France; 5 Department of Immunobiology, Yale University School of Medicine, New Haven, CT; 6 Howard Hughes Medical Institute, Chevy Chase, MD

## Abstract

Despite their importance in lung health and disease, it remains unknown how human alveolar macrophages develop early in life. Here we define the ontogeny of human alveolar macrophages from embryonic progenitors in vivo, using a humanized mouse model expressing human cytokines (MISTRG mice). We identified alveolar macrophage progenitors in human fetal liver that expressed the GM-CSF receptor CD116 and the transcription factor *MYB*. Transplantation experiments in MISTRG mice established a precursor–product relationship between CD34^−^CD116^+^ fetal liver cells and human alveolar macrophages in vivo. Moreover, we discovered circulating CD116^+^CD64^−^CD115^+^ macrophage precursors that migrated from the liver to the lung. Similar precursors were present in human fetal lung and expressed the chemokine receptor CX3CR1. Fetal CD116^+^CD64^−^ macrophage precursors had a proliferative gene signature, outcompeted adult precursors in occupying the perinatal alveolar niche, and developed into functional alveolar macrophages. The discovery of the fetal alveolar macrophage progenitor advances our understanding of human macrophage origin and ontogeny.

## Teaser

Evren et al. identify CD116^+^ fetal liver cells as precursors of human alveolar macrophages in early life and determine the impact of cell origin on lung macrophage identity and function in the human context with a unique in vivo model.

## Graphical Abstract

**Figure:**
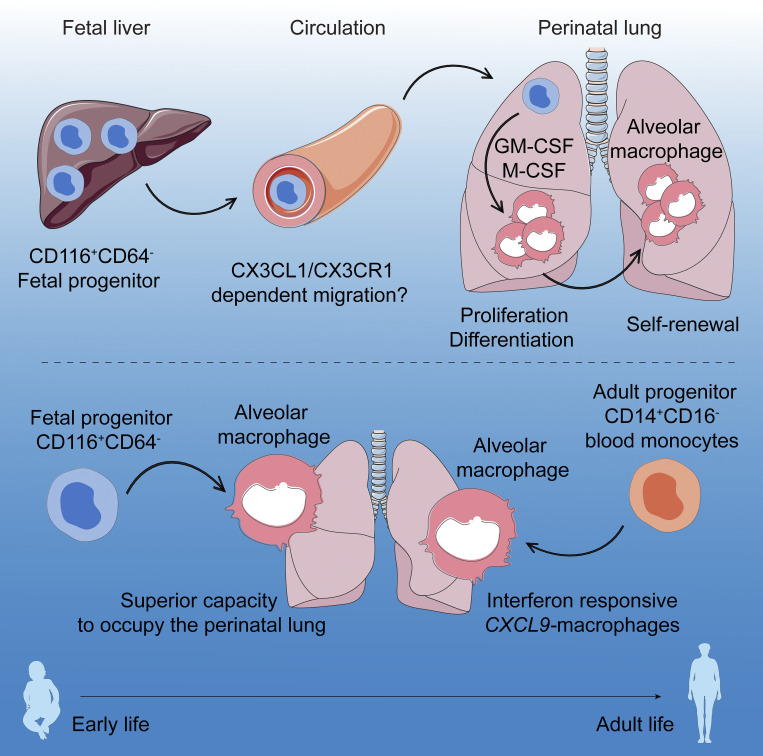

## Introduction

The lung is a vital organ that mediates the uptake of oxygen and is continuously exposed to inhaled microbes, particles, and allergens. Alveolar macrophages are the most abundant cell type in the airways and are essential for healthy lung function and barrier immunity (Evren et al., 2020; Garbi and Lambrecht, 2017; Hussell and Bell, 2014; Joshi et al., 2018; Kopf et al., 2015; Kulikauskaite and Wack, 2020; Puttur et al., 2019). They remove airborne microbes and cell debris from the airways through phagocytosis, which is essential to prevent harmful inflammation and to maintain the essential gas exchange. Another important function of alveolar macrophages is the catabolism of lung surfactant. Alveolar macrophages are critically dependent on the cytokine GM-CSF that is produced by alveolar epithelial cells (Dranoff et al., 1994; Gschwend et al., 2021; Guilliams et al., 2013; Schneider et al., 2014). Lack of GM-CSF and therefore alveolar macrophages results in pulmonary alveolar proteinosis (PAP) in both mice and humans due to the defective clearance of surfactant (Trapnell et al., 2019).

The cellular origin of macrophages varies depending on developmental age, organ of residence, and tissue state (Blériot et al., 2020; Guilliams and Svedberg, 2021; Guilliams et al., 2020; Jenkins and Allen, 2021). Studies in mice have demonstrated that macrophages arise either from embryonic progenitors or from blood monocytes that are derived from adult hematopoiesis (Epelman et al., 2014; Ginhoux and Guilliams, 2016; Perdiguero and Geissmann, 2016; Varol et al., 2015). Mouse alveolar macrophages are mostly of fetal origin in steady-state (Gomez Perdiguero et al., 2015; Guilliams et al., 2013; Hashimoto et al., 2013; Schneider et al., 2014; Yona et al., 2013; Yu et al., 2017) with increased contribution from blood monocytes during aging (Gomez Perdiguero et al., 2015; Liu et al., 2019), infection (Aegerter et al., 2020; Machiels et al., 2017; McCubbrey et al., 2018; Misharin et al., 2017; Mould et al., 2017; Mould et al., 2019), fibrosis (Aran et al., 2019; Joshi et al., 2020; McQuattie-Pimentel et al., 2021; Misharin et al., 2017), and lung regeneration (Lechner et al., 2017). Alveolar macrophages develop in humans after birth when the lungs are inflated by air uptake (Alenghat and Esterly, 1984; Bharat et al., 2016). Humans undergoing lung transplantation have an alveolar macrophage compartment that is of mixed origin, composed of both resident host macrophages and donor-derived macrophages originating from circulating monocytes (Bittmann et al., 2001; Byrne et al., 2020; Eguíluz-Gracia et al., 2016; Kjellström et al., 2000; Nayak et al., 2016). However, the ontogeny of human macrophages is poorly understood because invasive in vivo experiments are impossible in humans.

To overcome this limitation, we previously developed a humanized mouse model, named MISTRG, which expresses critical human factors for macrophages, such as GM-CSF and macrophage-CSF (M-CSF), through gene knock-in (Rongvaux et al., 2013; Rongvaux et al., 2014; Willinger et al., 2011a; Willinger et al., 2011b). MISTRG mice support human macrophage reconstitution after transplantation with human CD34^+^ hematopoietic stem and progenitor cells (HSPCs; Evren et al., 2021; Rongvaux et al., 2014; Saito et al., 2016; Sippel et al., 2019). This model therefore provides the opportunity to study the ontogeny of human macrophages in vivo (Alisjahbana et al., 2020; Evren et al., 2020).

We recently used MISTRG mice to dissect the development of human lung macrophages that are derived from blood monocytes (Evren et al., 2021). Specifically, we discovered that classical CD14^+^ blood monocytes derived from CD34^+^ HSPCs are the adult precursors of human alveolar macrophages. Blood monocyte-derived macrophages are most relevant in context of lung injury and inflammation. In early life and under physiological conditions, human alveolar macrophages are likely mostly of embryonic origin, i.e., independent of blood monocytes. We therefore now aimed to identify the fetal progenitor of human alveolar macrophages. We also examined the importance of cell origin on human lung macrophage specification by comparing the transcriptional signatures of human lung macrophages of fetal versus adult origin, as well as their ability to populate the perinatal niche, to self-renew, and to catabolize lung surfactant. We discovered that cell origin has an impact on alveolar niche occupation by macrophage precursors and on the functional identity of human lung macrophages. Specifically, we found that fetal macrophage precursors had a greater capacity to occupy the perinatal alveolar niche, whereas adult precursors generated IFN-responsive macrophages that are expanded in inflammatory lung diseases in humans, such as in severe coronavirus disease 2019 (COVID-19).

## Results

### Candidate progenitors of human lung macrophages are present in fetal liver

**Figure 1.**
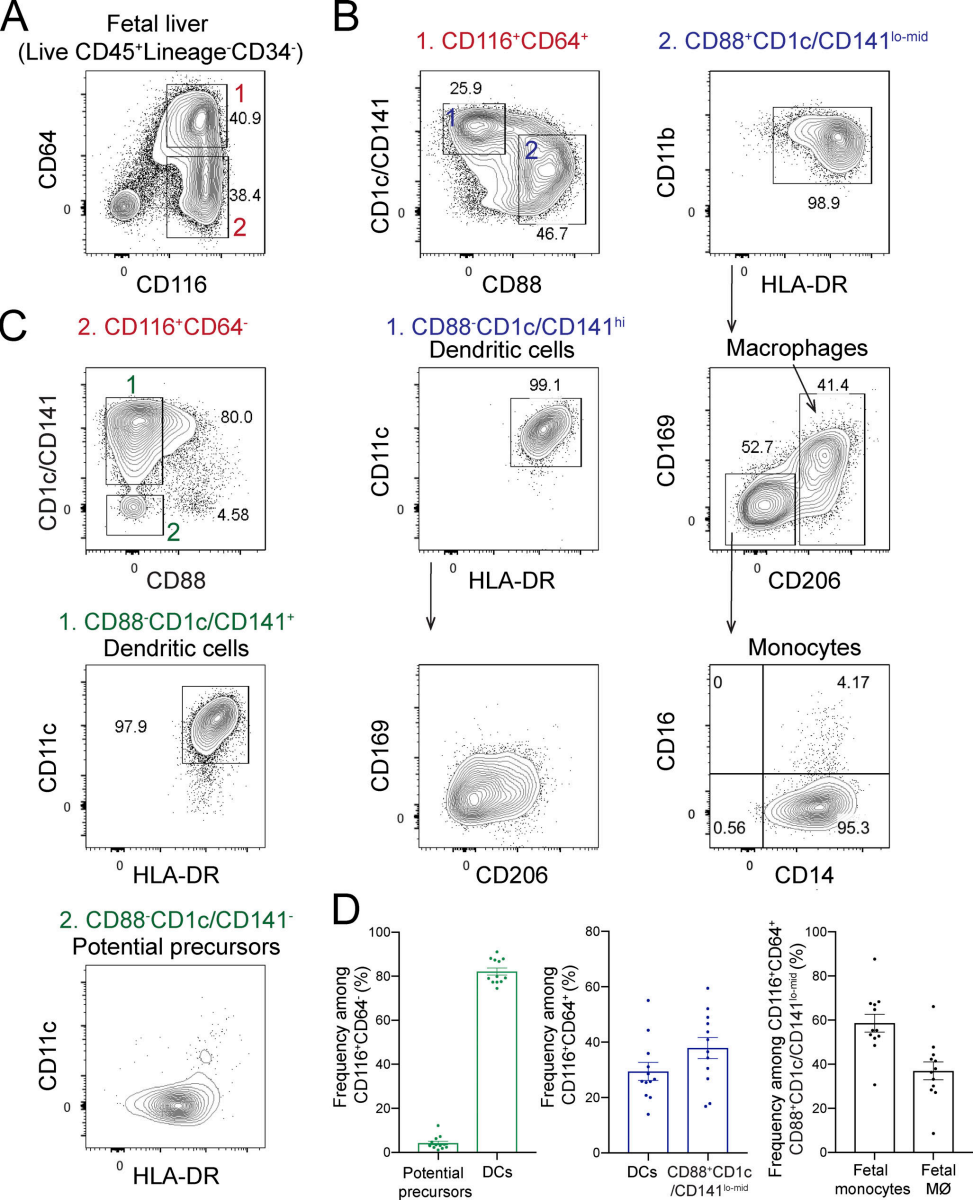
**Candidate alveolar macrophage progenitors are present in human fetal liver. (A)** Flow cytometry analysis of CD116-expressing cells within the CD34^−^ fraction of human fetal liver (17 wk of gestation). See Fig. S1 for gating strategy. After excluding cells expressing Lin markers (CD3, TCRαβ, TCRγδ, CD19, CD20, CD56, CD94, NKp46, and CD66abce), CD45^+^CD116^+^ cells were divided into CD64^+^ (population 1) and CD64^−^ (population 2) subsets. **(B)** Flow cytometry of CD116^+^CD64^+^ cells as gated in A. CD116^+^CD64^+^ cells were separated into CD88^−^CD1c/CD141^hi^ (population 1) and CD88^+^CD1c/CD141^lo-mid^ (population 2) subsets, corresponding to dendritic cells (CD11c^+^HLA-DR^hi^) and monocytes (CD11b^+^HLA-DR^+^CD206^−^CD169^−^) as well as macrophages (CD11b^+^HLA-DR^+^CD206^+^CD169^mid-hi^), respectively. **(C)** Flow cytometry of CD116^+^CD64^−^ cells as gated in A. Population 1 (CD88^−^CD1c/CD141^+^) contained CD11c^+^HLA-DR^hi^ dendritic cells. Population 2 (CD88^−^CD1c/CD141^−^) consisted of CD11c^−^HLA-DR^mid^ potential precursors. **(D)** Frequencies of the indicated populations in human fetal liver. DCs, dendritic cells; hi, high; lo-mid, low-mid; MØ, macrophages. Data are represented as mean ± SEM. Data (A–C) show one fetal liver sample (17 wk of gestation) representative of 12 samples from two independent experiments. Data (D) are pooled from two independent experiments with 12 fetal liver samples at 15–23 wk of gestation.

**Figure S1.**
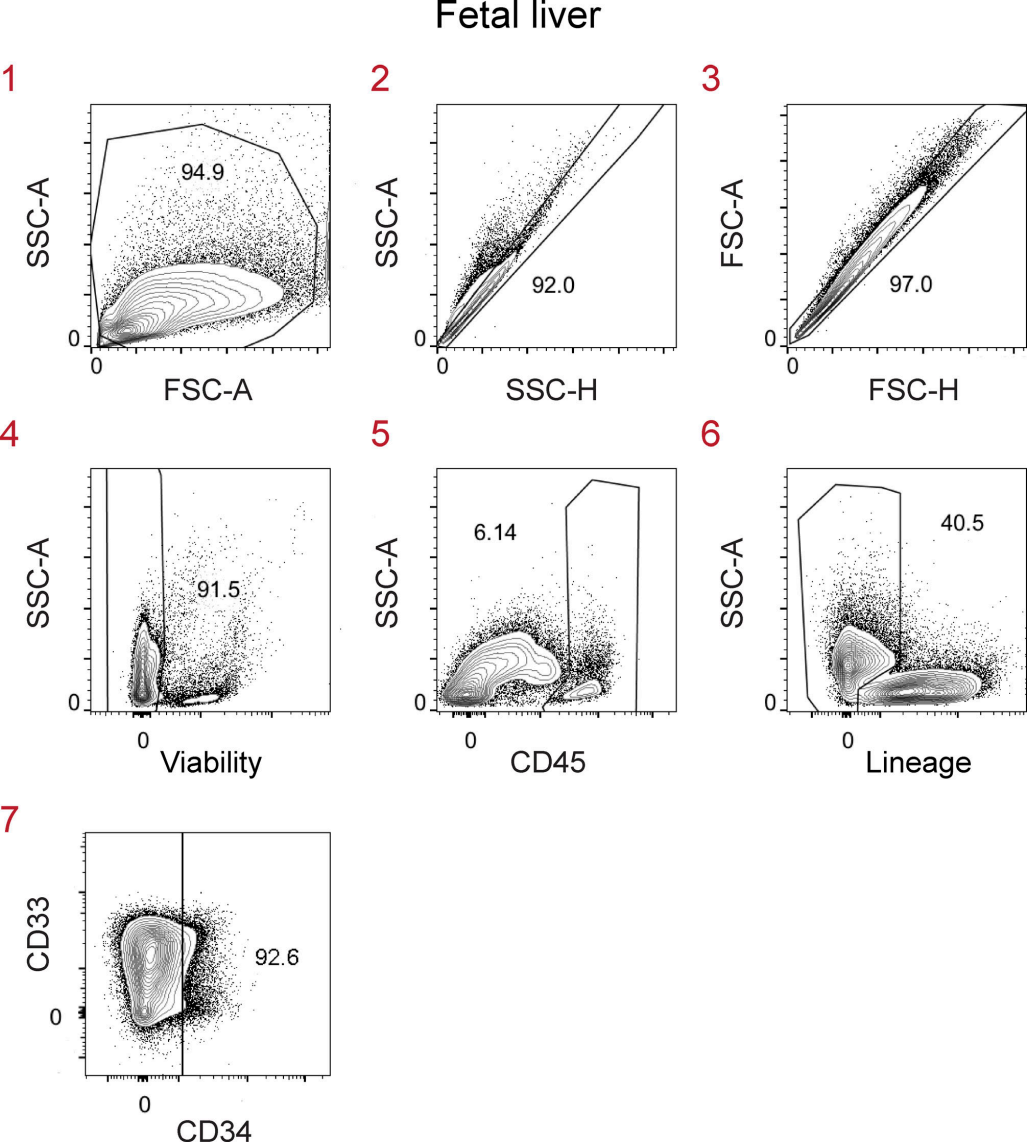
**Gating strategy to identify candidate macrophage precursors in human fetal liver.** Single-cell suspensions from fetal liver (17 wk gestation) were first gated on live CD45^+^Lin^−^CD34^−^ single cells before gating on CD116^+^ cells as shown in Fig. 1 A. Lin markers were CD3, TCRαβ, TCRγδ, CD19, CD20, CD56, CD94, NKp46, and CD66abce. FSC-A, forward scatter area; FSC-H, forward scatter height; SSC-A, side scatter area; SSC-H, side scatter height. Data are representative of two independent experiments with 12 fetal liver samples at 15–23 wk of gestation. The red numbers indicate the order of the flow cytometry plots (sequential gating of cells).

Due to the critical role of GM-CSF, we predicted that expression of GM-CSF receptor marks alveolar macrophage progenitors in human fetal tissue. Among fetal tissues, the liver is a hematopoietic organ (Cao et al., 2020; Park et al., 2020; Popescu et al., 2019) around the time of birth, when alveolar macrophages develop in mice (Guilliams et al., 2013; Yu et al., 2017). We therefore examined the presence of candidate precursors of alveolar macrophages in human fetal liver at the second trimester of gestation, the time of lung macrophage development in the human fetus (Dame et al., 1999). Consistent with our prediction, we identified CD45^+^CD34^−^ myeloid cells in fetal liver tissue that expressed the α-chain of the GM-CSF receptor (CD116) but did not express lineage (Lin) markers for T cells (CD3, TCRαβ, TCRγδ), B cells (CD19, CD20), natural killer cells (CD56, CD94, NKp46), and granulocytes (CD66abce; Fig. 1 A and Fig. S1). CD116^+^ myeloid cells consisted of CD64^+^ cells, containing fetal dendritic cells (CD88^−^CD1c/CD141^hi^CD11c^+^HLA-DR^hi^), monocytes (CD88^+^CD1c/CD141^lo/mid^CD11b^+^HLA-DR^+^CD206^−^CD169^−^CD14^+^), and macrophages (CD88^+^CD1c/CD141^lo/mid^CD11b^+^HLA-DR^+^CD206^+^CD169^+^; Fig. 1 B), as well as of CD64^−^ cells comprising dendritic cells and putative precursor-like cells (CD88^−^CD1c/CD141^−^CD11c^−^HLA-DR^mid^; Fig. 1, C and D). In summary, these data identify GM-CSF receptor-expressing candidate precursors of human lung macrophages in the fetal liver.

### CD116^+^CD64^−^ fetal precursor-like cells express *MYB* and have a proliferative gene signature

**Figure 2.**
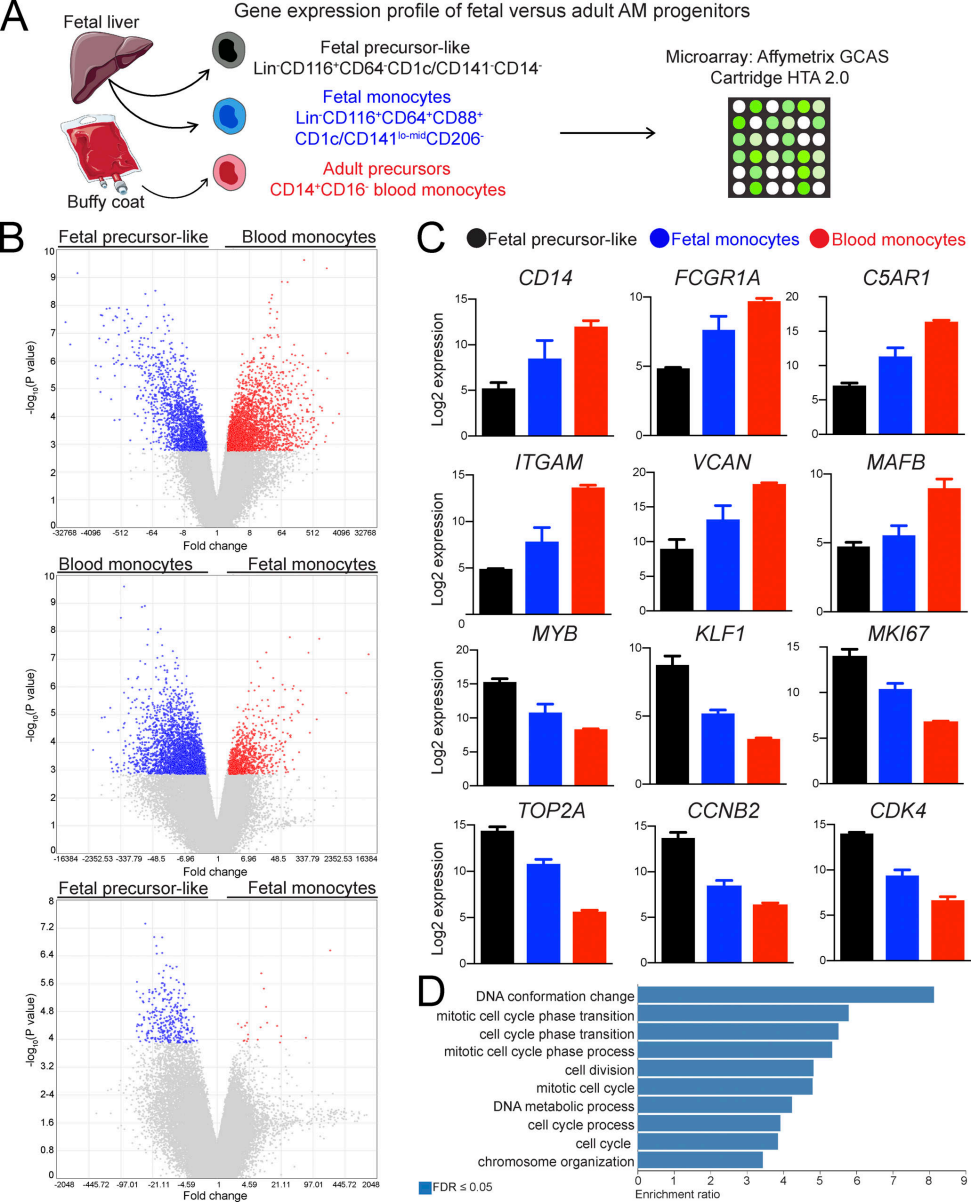
**Gene signatures of CD116^+^CD64^−^ fetal precursor-like cells, CD116^+^CD64^+^ fetal monocytes, and adult CD14^+^CD16^−^ blood monocytes. (A)** Experimental outline to define the gene expression profiles of the indicated human cell populations. Cartoon was adapted from Servier Medical Art. **(B)** Volcano plots of differentially expressed genes (DEGs) between the indicated cell populations. Fold change is plotted versus -log_10_ P value (not corrected for multiple testing). DEGs with a fold change ≥2 and an FDR-corrected P value ≤0.05 are highlighted in blue and red. **(C)** Bar graphs showing expression of selected genes in CD116^+^CD64^−^ fetal precursor-like cells, CD116^+^CD64^+^ fetal monocytes, and adult CD14^+^CD16^−^ blood monocytes. Data are represented as mean ± SEM. **(D)** Gene ontology over-representation analysis of DEGs up-regulated in CD116^+^CD64^−^ fetal precursor-like cells compared with adult CD14^+^CD16^−^ blood monocytes and CD116^+^CD64^+^ fetal monocytes. AM, alveolar macrophages; GCAS, GeneChip Array Station; HTA, Human Transcriptome Array; lo-mid, low-mid. Data (B–D) are from a single microarray experiment with three replicates per cell population (isolated from individual fetal liver or blood samples) obtained from four independent cell sorting experiments.

To gain further insights into their distinctive features, we determined the gene expression profiles of fetal candidate progenitors of human alveolar macrophages by microarray (Fig. 2, A and B). For this purpose, potential fetal precursors of human alveolar macrophages among CD45^+^CD34^−^Lin^−^CD116^+^ cells were isolated from fetal liver. Specifically, we purified CD64^+^CD88^+^CD1c/CD141^lo-mid^CD206^−^CD169^−^ fetal monocytes, described as precursors of mouse alveolar macrophages (Guilliams et al., 2013; Schneider et al., 2014; Yu et al., 2017), and precursor-like fetal cells that lacked expression of monocyte (CD14, CD64) and dendritic cell markers (CD1c, CD141). We then compared their gene expression profiles to that of CD14^+^CD16^−^ blood monocytes, adult precursors of human alveolar macrophages (Evren et al., 2021). Consistent with their cellular identity, CD14^+^CD16^−^ blood monocytes highly expressed monocytic genes, such as *CD14*, *FCGR1A* (encoding CD64), *ITGAM* (encoding CD11b), *C5AR1* (encoding CD88), *VCAN*, *CSF3R*, and *LYZ*, but these genes were also detected in fetal CD116^+^CD64^−^ precursor-like cells (Fig. 2 C and Table S1). In contrast, fetal CD116^+^CD64^−^ precursor-like cells had higher mRNA expression of the transcription factor *MYB* (Fig. 2 C and Table S1) that is expressed in HSPCs and myeloid progenitors (Bian et al., 2020). In addition, fetal CD116^+^CD64^−^ precursor-like cells expressed less *MAFB* (Fig. 2 C and Table S1), a transcription factor that antagonizes MYB and represses macrophage self-renewal (Aziz et al., 2009; Li et al., 2020; Soucie et al., 2016). Furthermore, fetal CD116^+^CD64^−^ precursor-like cells expressed genes and transcription factors (*GATA1*, *KLF1*, *TAL1*) associated with erythrocyte differentiation (Popescu et al., 2019), consistent with preferential erythroid-myeloid hematopoiesis in human fetal liver (Jardine et al., 2021). Therefore, fetal CD116^+^CD64^−^ precursor-like cells resembled *Myb*-expressing erythro-myeloid progenitors (EMPs) and EMP-derived CD64^−^ myeloid progenitors that further differentiate into CD64^+^ fetal monocytes and alveolar macrophages in mice (Gomez Perdiguero et al., 2015; Hoeffel et al., 2015; Li et al., 2020; Mass et al., 2016). Moreover, fetal CD116^+^CD64^−^ precursor-like cells preferentially expressed *MYC* and other genes promoting cell division and proliferation, such as *MKI67*, *TOP2A*, *PCNA*, cyclins, and cyclin-dependent kinases (Fig. 2 C and Table S1), similar to what has been reported for human fetal liver progenitors (Popescu et al., 2019). Global analysis confirmed that genes related to cell cycle and cell division were overrepresented in fetal CD116^+^CD64^−^ cells when compared with both adult and fetal monocytes (Fig. 2 D). These findings indicate that fetal CD116^+^CD64^−^ precursor-like cells have high proliferative capacity and resemble fetal progenitors of mouse alveolar macrophages.

### CD116-expressing fetal liver cells have human alveolar macrophage potential in vivo

**Figure 3.**
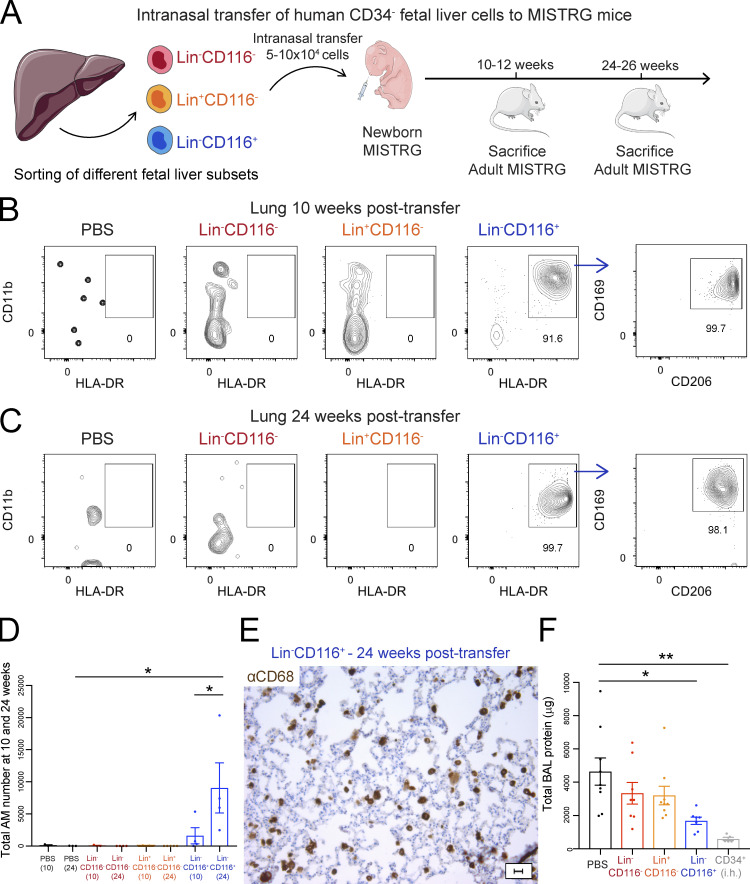
**CD116-expressing fetal liver cells generate human alveolar macrophages in vivo. (A)** Intranasal transfer of purified human Lin^−^CD116^−^, Lin^+^CD116^−^, and Lin^−^CD116^+^ populations from CD34^−^ fetal liver cells into newborn MISTRG mice. Lin markers were CD3, CD19, CD56, NKp46, and CD66abce. Control mice received PBS only. Cartoon was adapted from Servier Medical Art. **(B and C)** Flow cytometry of human CD45^+^CD11b^+^HLA-DR^+^CD206^+^CD169^+^ macrophages in lung tissue of MISTRG mice at 10 wk (B) and 24 wk (C) after transfer of the different fetal liver cell subsets. See Fig. S2 A for gating strategy. **(D)** Number of human alveolar macrophages (AM) in MISTRG mice at 10 and 24 wk after transplantation. **(E)** Immunohistochemistry of lung sections from MISTRG mice 24 wk after transplantation with human Lin^−^CD116^+^ fetal liver cells. Lung sections were stained with anti-human CD68 antibody (brown). Scale bar is 20 µm. **(F)** Amounts of total protein in the BAL fluid of MISTRG mice 24 wk after transplantation with human fetal liver cells. Control mice received PBS only or were transplanted with human CD34^+^ HSPCs from cord blood by intrahepatic injection (i.h.). aCD68, anti-CD68. Data are represented as mean ± SEM. *, P < 0.05; **, P < 0.01 (one-way ANOVA with Tukey’s multiple comparison post hoc test). Data (B and C) show one lung sample representative of three to five samples (individual mice) per group and time point from three independent experiments. Data (E) show one lung sample representative of four samples (individual mice) from three independent experiments. Data (D and F) are pooled from three independent experiments with *n* = 3–5 per group and time point (D) or *n* = 5–9 per group (F).

**Figure S2.**
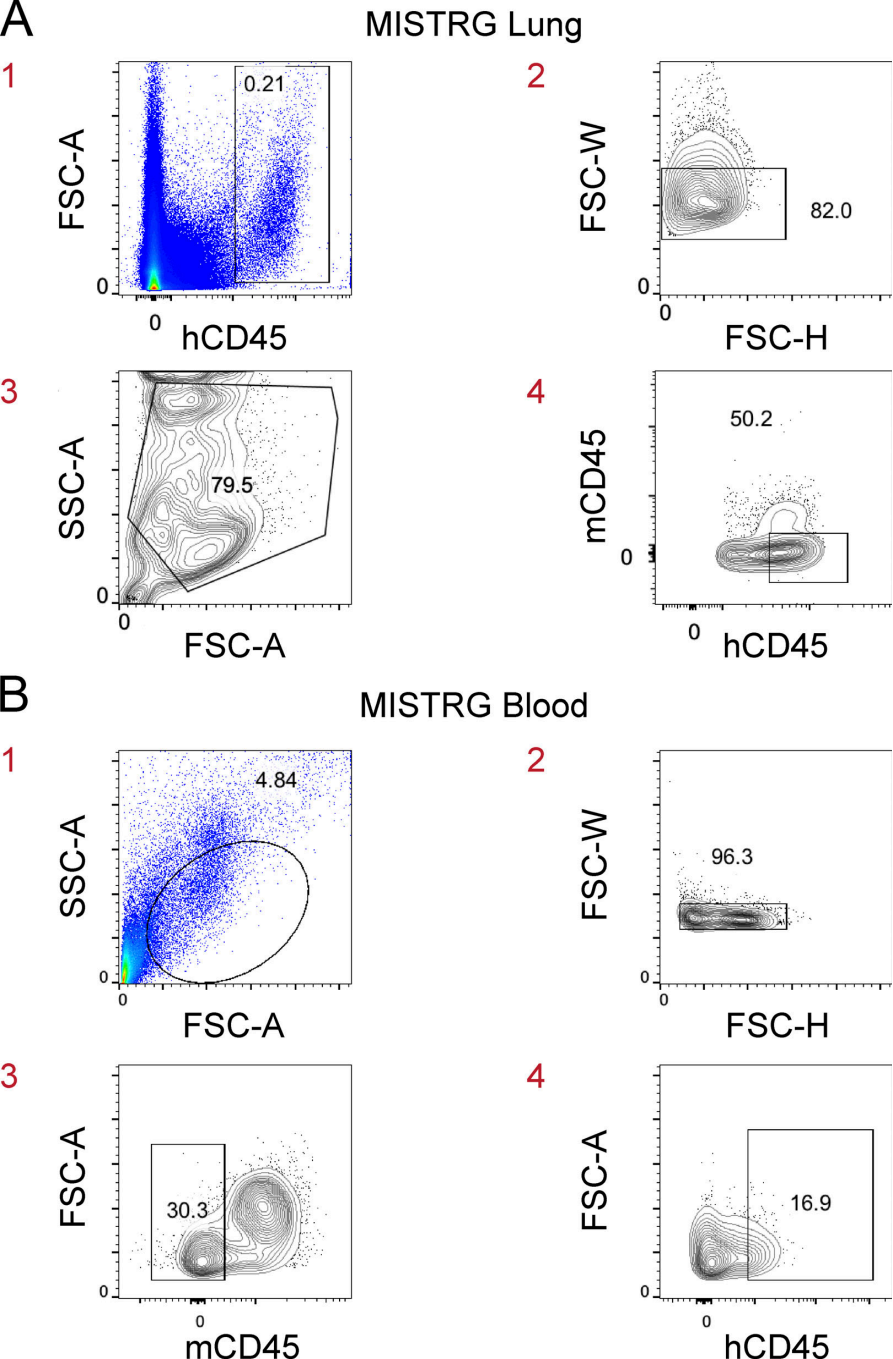
**Gating strategy to identify human hematopoietic cells in MISTRG mice. (A)** Single-cell suspensions from the lung of MISTRG mice were gated on human CD45^+^ single cells before gating on human lung macrophages in Fig. 3, B and C, Fig. 4, B and C, and Fig. 5 C. hCD45, human CD45; mCD45, mouse CD45. **(B)** Blood cells from MISTRG mice were gated on human CD45^+^ single cells before gating on circulating human macrophage precursors in Fig. 4 G and Fig. 6 A. FSC-A, forward scatter area; FSC-H, forward scatter height; FSC-W, forward scatter width; hCD45, human CD45; mCD45, mouse CD45; SSC-A, side scatter area. Data (A) show one lung sample representative of three to five samples (individual mice) from three independent experiments. Data (B) show one blood sample representative of seven to nine samples (individual mice) from two independent experiments. The red numbers indicate the order of the flow cytometry plots (sequential gating of cells).

**Figure S3.**
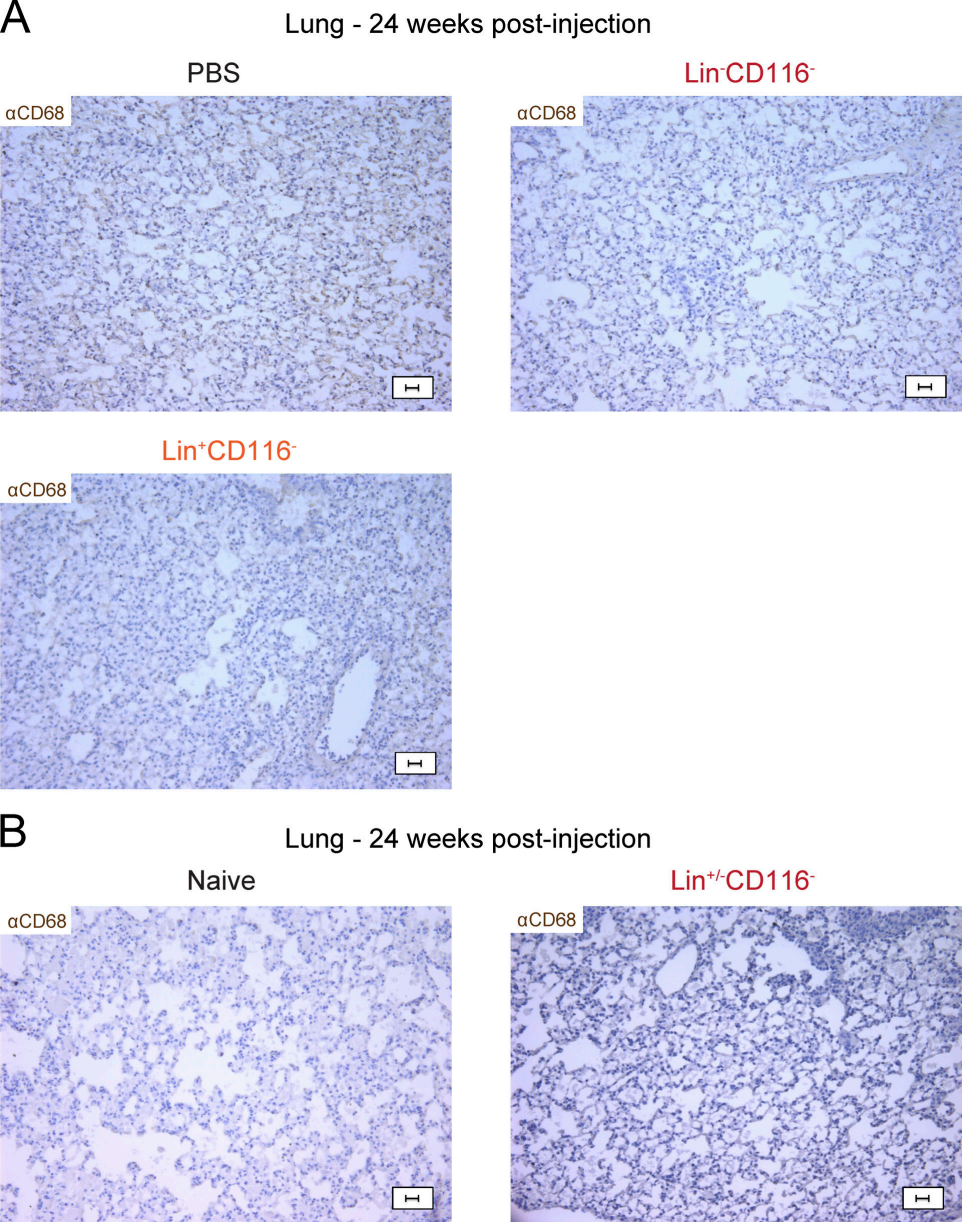
**Controls for immunohistochemistry of lung sections. (A and B)** Immunohistochemistry of lung sections from MISTRG mice 24 wk after intranasal (A) or intrahepatic (B) transplantation with the indicated cell populations (as in Fig. 3 E and Fig. 4 E). Control mice received PBS or no cells (naive). Lung sections were stained with anti-human CD68 antibody (brown). Scale bars are 20 µm. aCD68, anti-CD68. Data (A and B) show one lung sample representative of three to four samples (individual mice) from three independent experiments (A) or representative of two samples (individual mice) from two independent experiments (B).

Having identified candidate progenitor populations, we tested their ability to differentiate into lung macrophages in vivo. For this purpose, we performed transplantation experiments to establish precursor-product relationships. MISTRG mice are well-suited for this approach since (1) the empty alveolar niche (lack of mouse GM-CSF and therefore mouse alveolar macrophages) allows transplanted progenitors to colonize the niche; and (2) the expression of human GM-CSF in the alveolar space supports the differentiation into mature human alveolar macrophages (Evren et al., 2021; Rongvaux et al., 2014; Willinger et al., 2011b). We transplanted fetal liver populations into the airways of newborn MISTRG recipient mice, as the first week of life is the physiological time when the alveolar niche becomes colonized in mice (Guilliams et al., 2013). We first assessed the ability of fetal CD34^−^Lin^−^CD116^+^ candidate progenitors compared with that of CD34^−^Lin^−^CD116^−^ and CD34^−^Lin^+^CD116^+^ cells to reconstitute the alveolar niche after intranasal transplantation (Fig. 3 A). Transplanted CD34^−^Lin^−^CD116^+^ fetal liver cells colonized the lung and differentiated into human macrophages with an alveolar macrophage phenotype (CD11b^+^HLA-DR^+^CD206^+^CD169^+^; Bharat et al., 2016; Desch et al., 2016; Yu et al., 2016) within 10 wk of transfer and were retained at 24 wk, whereas neither CD34^−^Lin^−^CD116^−^ nor CD34^−^Lin^+^CD116^−^ cells were able to do so (Fig. 3, B–D; and Fig. S2 A). Human CD68^+^ macrophages derived from CD34^−^Lin^−^CD116^+^ cells could be visualized in the alveoli by immunohistochemistry, confirming their correct physiological localization (Fig. 3 E and Fig. S3 A).

**Figure S4.**
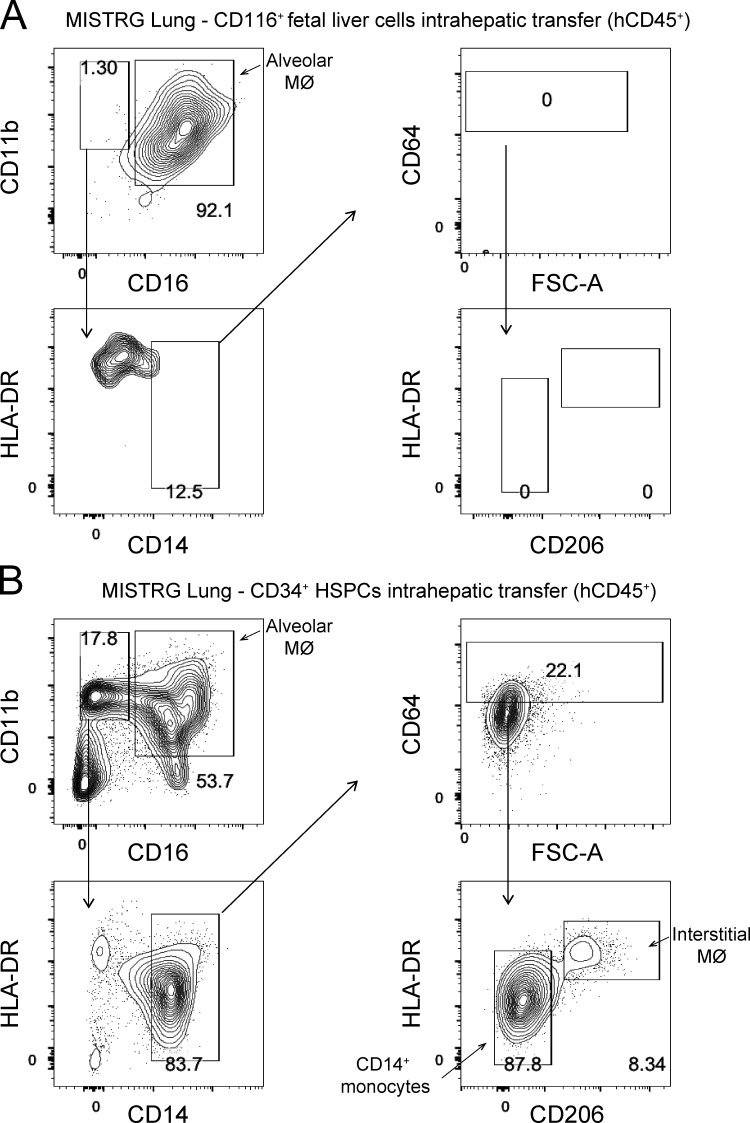
**Human interstitial lung macrophages do not develop in MISTRG mice transplanted with CD116^+^ fetal liver cells.** **(A and B)** Flow cytometry of human interstitial macrophages in lung tissue from MISTRG mice 24 wk after transplantation with human CD116^+^ fetal liver cells (A) or CD34^+^ HSPCs (B). Interstitial lung macrophages were gated as CD45^+^CD11b^+^CD14^+^CD16^−^CD64^+^CD206^+^HLA-DR^+^ cells. FSC-A, forward scatter area; hCD45, human CD45; MØ, macrophages. Data (A and B) show one lung sample representative of three to five samples (individual mice) per group from three independent experiments.

We also examined whether human interstitial lung macrophages were present in MISTRG mice transplanted with fetal CD34^−^Lin^−^CD116^+^ cells, using a recently described gating strategy for human interstitial macrophages (Chakarov et al., 2019). Flow cytometry showed that the lungs of MISTRG mice transplanted with fetal CD34^−^Lin^−^CD116^+^ cells did not harbor any cells with a surface phenotype (CD11b^+^CD64^+^CD14^+^CD16^−^CD206^+^CD169^+^HLA-DR^hi^) that is characteristic of human interstitial macrophages (Fig. S4 A). In contrast, human interstitial lung macrophages developed in MISTRG mice transplanted with human CD34^+^ HSPCs (Fig. S4 B) as we reported previously (Evren et al., 2021). These data suggest that the developmental potential of CD116^+^ fetal precursors in the lung may be restricted to becoming alveolar macrophages.

To test the function of fetal progenitor-derived macrophages, we determined their capacity to prevent PAP. We previously showed that nontransplanted MISTRG mice lack mouse alveolar macrophages and develop PAP at ∼4 wk of age, which can be prevented by the transplantation with human CD34^+^ HSPCs (Evren et al., 2021; Willinger et al., 2011b). MISTRG mice transplanted with fetal CD34^−^Lin^−^CD116^+^ cells had lower amounts of protein in bronchoalveolar lavage (BAL) fluid than nontransplanted MISTRG mice and mice transplanted with CD34^−^Lin^−^CD116^−^ or CD34^−^Lin^+^CD116^−^ cells, indicating PAP rescue (Fig. 3 F). Therefore, human alveolar macrophages arising from fetal CD34^−^Lin^−^CD116^+^ cells were functional since they were able to catabolize lung surfactant. Combined, these results establish a precursor–product relationship between fetal CD34^−^Lin^−^CD116^+^ cells and human alveolar macrophages.

### Fetal CD116^+^ alveolar macrophage progenitors are able to migrate from the liver to the lung

**Figure 4.**
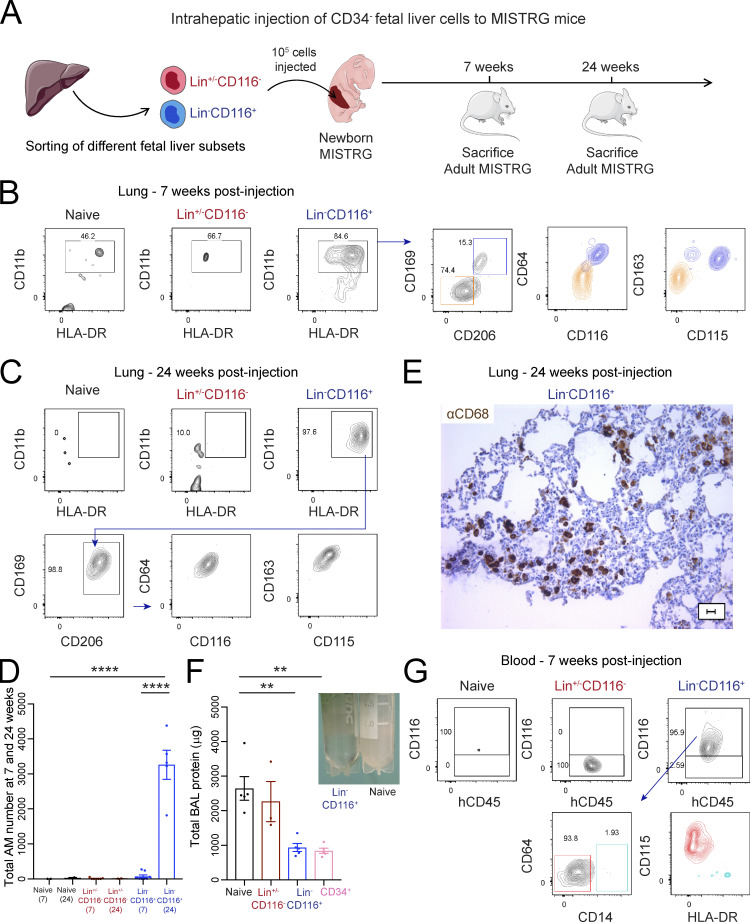
**CD116^+^ fetal liver cells are able to migrate to the lung and differentiate into human alveolar macrophages. (A)** Intrahepatic injection of purified human Lin^+/−^CD116^−^ and Lin^−^CD116^+^ populations from CD34^−^ fetal liver cells into newborn MISTRG mice. Lin markers were CD3, CD19, CD56, NKp46, and CD66abce. Cartoon was adapted from Servier Medical Art. **(B and C)** Flow cytometry of MISTRG lungs 7 wk (B) and 24 wk (C) after injection of human cells. Control mice were not injected with cells (naive). See Fig. S2 A for gating strategy. **(D)** Number of human alveolar macrophages (AM) in MISTRG mice at 10 and 24 wk after transplantation. **(E)** Immunohistochemistry of lung sections from MISTRG mice 24 wk after transplantation with human Lin^−^CD116^+^ fetal liver cells. Lung sections were stained with anti-human CD68 antibody (brown). Scale bar is 20 µm. **(F)** Amounts of total protein in the BAL fluid of MISTRG mice 24 wk after transplantation with human Lin^-/+^CD116^−^ or Lin^−^CD116^+^ fetal liver cells. Control mice were transplanted with human CD34^+^ HSPCs from cord blood or were not injected with cells (naive). Data are represented as mean ± SEM. Picture on the right shows BAL fluid obtained from MISTRG mice transplanted with Lin^−^CD116^+^ fetal liver cells or not transplanted with any human cells (naive). **(G)** Flow cytometry of MISTRG blood 7 wk after transplantation with the indicated human cells. Control mice were not injected with cells (naive). See Fig. S2 B for gating strategy. aCD68, anti-CD68; hCD45, human CD45. Data are represented as mean ± SEM. **, P < 0.01; ****, P < 0.0001 (one-way ANOVA with Tukey’s multiple comparison post hoc test). Data (B and C) show one lung sample representative of two to eight samples (individual mice) per group and time point from three independent experiments. Data (E) show one lung sample representative of five samples (individual mice) from three independent experiments. Data (D and F) are pooled from three independent experiments with *n* = 2–8 per group and time point (D) or *n* = 3–5 per group (F). Data (G) show one blood sample representative of seven to nine samples (individual mice) from two independent experiments.

We next predicted that fetal CD34^−^Lin^−^CD116^+^ lung macrophage progenitors traffic from the liver to the lung. Therefore, instead of transplanting fetal liver progenitors into the airways, we injected them into newborn MISTRG mice via the intrahepatic route to track their migration from the liver to the lung (Fig. 4 A). Consistent with our prediction, flow cytometry showed that alveolar macrophages (CD11b^+^HLA-DR^+^CD206^+^CD169^+^CD64^+^CD163^+^) populated the lungs of MISTRG mice 7 wk after intrahepatic injection of fetal CD34^−^Lin^−^CD116^+^ cells and had abundant M-CSF receptor (CD115) surface expression (Fig. 4 B and Fig. S2 A). This observation suggested that fetal macrophage precursors are highly responsive to M-CSF, the major factor driving macrophage proliferation. Fetal progenitor-derived human alveolar macrophages had expanded in MISTRG mice when analyzed at 24 wk after transplantation (Fig. 4, C and D). Immunohistochemistry confirmed the presence of human CD68^+^ macrophages in the lungs of MISTRG mice (Fig. 4 E and Fig. S3 B). Finally, intrahepatic transplantation with fetal CD34^−^Lin^−^CD116^+^ cells alleviated PAP, as shown by lower amounts of BAL protein, similar to mice transplanted with CD34^+^ HSPCs (Fig. 4 F). To further characterize circulating lung macrophage progenitors, we examined the blood of MISTRG mice after transplantation with CD116^+^ fetal liver cells (Fig. 4 G and Fig. S2 B). The blood of MISTRG mice transplanted with fetal CD34^−^Lin^−^CD116^+^ cells contained CD116^+^CD64^−^ cells expressing CD115, but not CD14 (Fig. 4 G), identifying them as potential circulating alveolar macrophage precursors derived from the transplanted fetal CD34^−^Lin^−^CD116^+^ cells. Furthermore, this finding revealed that these potential CD116^+^CD64^−^ fetal alveolar macrophage precursors in the circulation were distinct from HSPC-derived CD14^+^CD116^+^ blood monocytes, which are adult precursors of human alveolar macrophages, as we showed recently (Evren et al., 2021). Taken together, we demonstrate that macrophage progenitors in the fetal liver enter the circulation and migrate from the blood into the lung, where they develop into human alveolar macrophages.

### CD116^+^CD64^−^ fetal liver cells generate mature human alveolar macrophages in vivo

**Figure 5.**
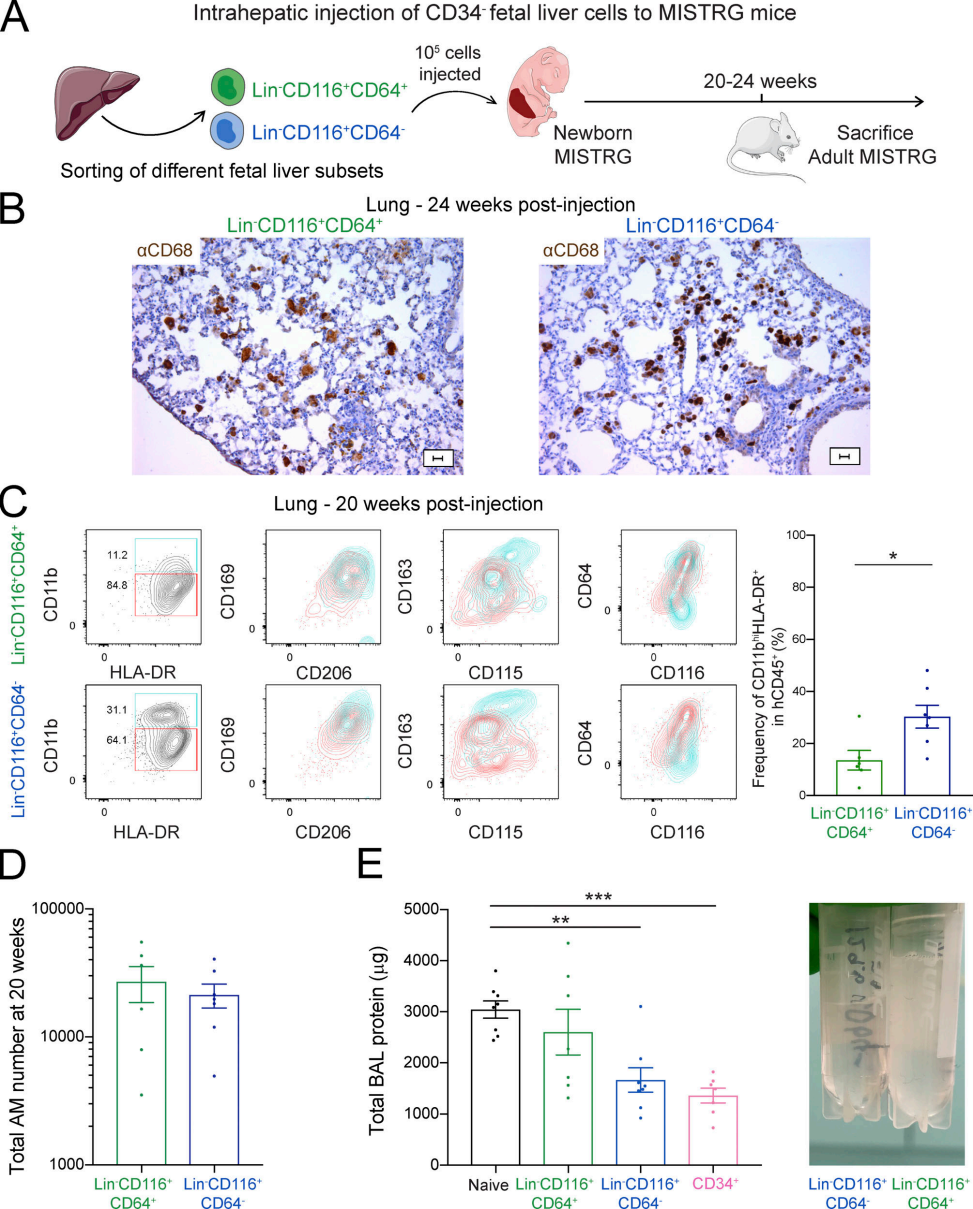
**CD116^+^CD64^−^ fetal liver cells give rise to human alveolar macrophages in vivo. (A)** Intrahepatic injection of purified human Lin^−^CD116^+^CD64^−^ and Lin^−^CD116^+^CD64^+^ populations from CD34^−^ fetal liver cells into newborn MISTRG mice. Lin markers were CD3, CD19, CD56, NKp46, and CD66abce. Cartoon was adapted from Servier Medical Art. **(B)** Immunohistochemistry of lung sections from MISTRG mice 24 wk after transplantation with either human Lin^−^CD116^+^CD64^−^ or Lin^−^CD116^+^CD64^+^ fetal liver cells. Lung sections were stained with anti-human CD68 antibody (brown). Scale bars are 20 µm. **(C)** Flow cytometry of human CD45^+^CD11b^+^HLA-DR^+^CD206^+^CD169^+^ lung macrophages in MISTRG mice 20 wk after transplantation. See Fig. S2 A for gating strategy. **(D)** Number of human alveolar macrophages (AM) derived from Lin^−^CD116^+^CD64^−^ and Lin^−^CD116^+^CD64^+^ fetal liver cells in MISTRG mice at 20 wk after transplantation. **(E)** Amounts of total protein in the BAL fluid of MISTRG 20–24 wk after transplantation with human Lin^−^CD116^+^CD64^−^ or Lin^−^CD116^+^CD64^+^ fetal liver cells. Newborn control MISTRG mice were either not transplanted or transplanted with human CD34^+^ HSPCs from cord blood. Picture on the right shows BAL fluid obtained from MISTRG mice transplanted with either Lin^−^CD116^+^CD64^−^ or Lin^−^CD116^+^CD64^+^ fetal liver cells. aCD68, anti-CD68; hCD45, human CD45; hi, high. Data are represented as mean ± SEM. *, P < 0.05 (unpaired Student’s *t* test), or **, P < 0.01; ***, P < 0.001 (one-way ANOVA with Tukey’s multiple comparison post hoc test). Data (B) show one lung sample representative of six to seven samples (individual mice) per group from two independent experiments. Data (C) show one lung sample representative of six to seven samples (individual mice) per group from two independent experiments. Data (C–E) are pooled from two independent experiments with *n* = 6 or 7 (C and D) or *n* = 7 or 8 (E) per group.

Having shown that alveolar macrophage progenitor activity is present within CD34^−^Lin^−^CD116^+^ fetal liver cells and that they generate putative circulating CD116^+^CD64^−^ precursors (Fig. 4 G), we further fractionated the progenitor activity of CD34^−^Lin^−^CD116^+^ fetal liver cells. Specifically, we compared the macrophage potential of CD116^+^CD64^−^ to that of CD116^+^CD64^+^ fetal liver cells after intrahepatic injection into newborn MISTRG mice (Fig. 5 A). Both populations gave rise to human CD68^+^ lung macrophages (Fig. 5 B) with an alveolar macrophage phenotype (CD11b^+^HLA-DR^+^CD206^+^CD169^+^CD64^+^) with similar efficiency (Fig. 5, C and D). Further analysis revealed that CD116^+^CD64^−^ fetal progenitors more efficiently generated CD11b^hi^CD163^hi^ macrophages, representing mature alveolar macrophages (Bharat et al., 2016) than CD116^+^CD64^+^ fetal progenitors (Fig. 5 C). Consistent with this observation, human macrophages derived from CD116^+^CD64^−^ fetal liver cells had the capacity to catabolize lung surfactant, a key function of alveolar macrophages, and to rescue PAP syndrome in MISTRG mice (Fig. 5 E). In contrast, macrophages arising from CD116^+^CD64^+^ fetal liver cells were unable to prevent PAP (Fig. 5 E).

**Figure 6.**
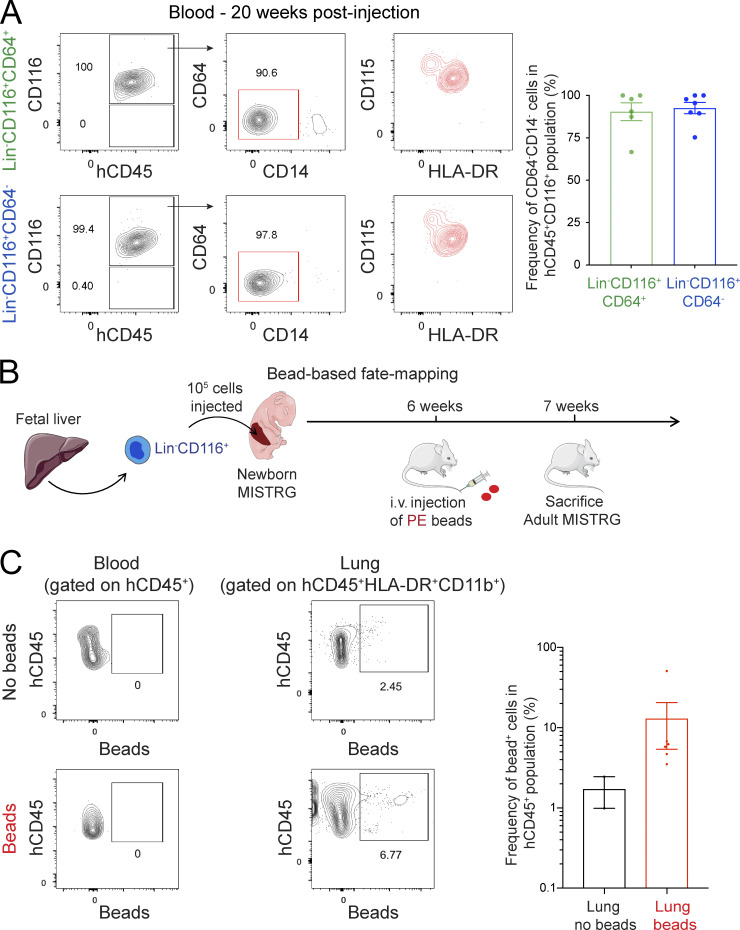
**Circulating CD116^+^CD64^−^CD14^−^CD115^+^ macrophage precursors migrate to the lung. (A)** Flow cytometry of MISTRG blood 20 wk after transplantation with human Lin^−^CD116^+^CD64^−^ or Lin^−^CD116^+^CD64^+^ fetal liver cells. See Fig. S2 B for gating strategy. Frequencies of circulating CD45^+^CD116^+^CD64^−^CD14^−^ cells after transplantation are shown on the right. **(B)** Fate-mapping of circulating cells in MISTRG mice transplanted with human Lin^−^CD116^+^ fetal liver cells. Blood cells were labeled by the i.v. injection of PE-conjugated fluorescent beads. Cartoon was adapted from Servier Medical Art. **(C)** Flow-cytometric analysis and frequency of bead^+^ cells in the lung and blood of MISTRG mice at day 7 after bead injection. hCD45, human CD45. Data are represented as mean ± SEM. Data (A) show one blood sample representative of six or seven samples (individual mice) per group from two independent experiments. Data (C) show one blood and lung sample representative of two to six samples per group from two independent experiments. Bar graphs (A and C) show data pooled from two independent experiments with *n* = 6–7 (A) or *n* = 2–6 (C) per group.

Analysis of blood demonstrated the presence of circulating CD116^+^CD64^−^CD14^−^CD115^+^HLA-DR^mid^ human cells in MISTRG mice after transplantation with CD116^+^CD64^+^ or CD116^+^CD64^−^ fetal liver cells (Fig. 6 A). CD116^+^CD64^−^CD14^−^ cells found in the blood of MISTRG mice had the same surface phenotype as CD116^+^CD64^−^ fetal liver cells that give rise to human alveolar macrophages after transplantation into MISTRG mice. Therefore, their phenotypic similarity indicated that CD116^+^CD64^−^CD14^−^CD115^+^HLA-DR^mid^ cells in the blood likely corresponded to circulating alveolar macrophage precursors in transit from the liver to the lung. To further corroborate this notion, we performed bead-based fate-mapping of circulating human hematopoietic cells in MISTRG mice transplanted with CD116^+^ fetal liver cells (Fig. 6 B) as in our recent study (Evren et al., 2021). 1 wk after the i.v. injection of fluorescent beads, we could detect CD11b^+^HLA-DR^+^ cells in the lung of MISTRG mice, derived from circulating CD116^+^CD64^−^CD14^−^ cells that had captured the beads and migrated into the lung tissue, as bead^+^ cells were not present in the blood anymore after 1 wk (Fig. 6 C). The bead^+^ cells in the lung therefore likely represented developing alveolar macrophages (Evren et al., 2021). Taken together, we conclude that CD64^−^ fetal liver cells expressing CD116 are likely the main progenitor of mature human alveolar macrophages.

**Figure 7.**
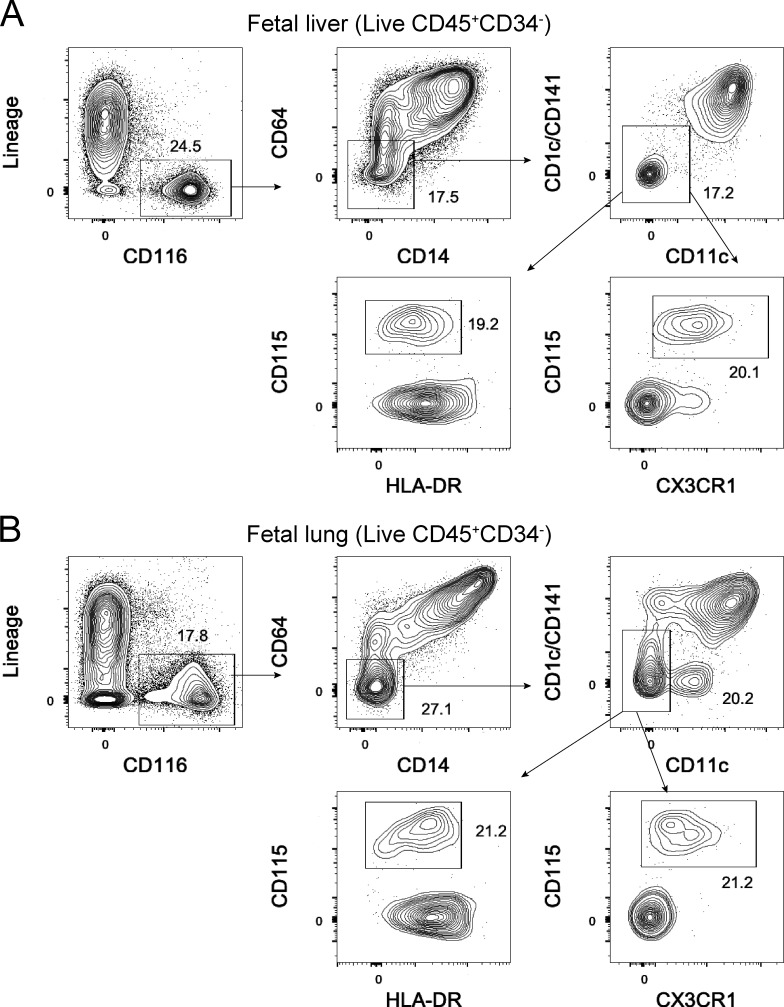
**Human fetal liver and lung contain CD116^+^CD64^−^CD115^+^CX3CR1^+^ lung macrophage precursors. (A and B)** Flow cytometry analysis of CD116-expressing macrophage precursors in human fetal liver (A) and fetal lung (B) at wk 21 and 22 of gestation, respectively. After pregating on CD45^+^CD34^−^ cells (see Fig. S5 for gating strategy), macrophage precursors were gated as shown. Lin markers were CD3, TCRαβ, TCRγδ, CD19, CD20, CD56, CD94, NKp46, and CD66abce. Data (A and B) are representative of fetal liver and fetal lung samples at 15–23 wk of gestation from two independent experiments (*n* = 12).

**Figure S5.**
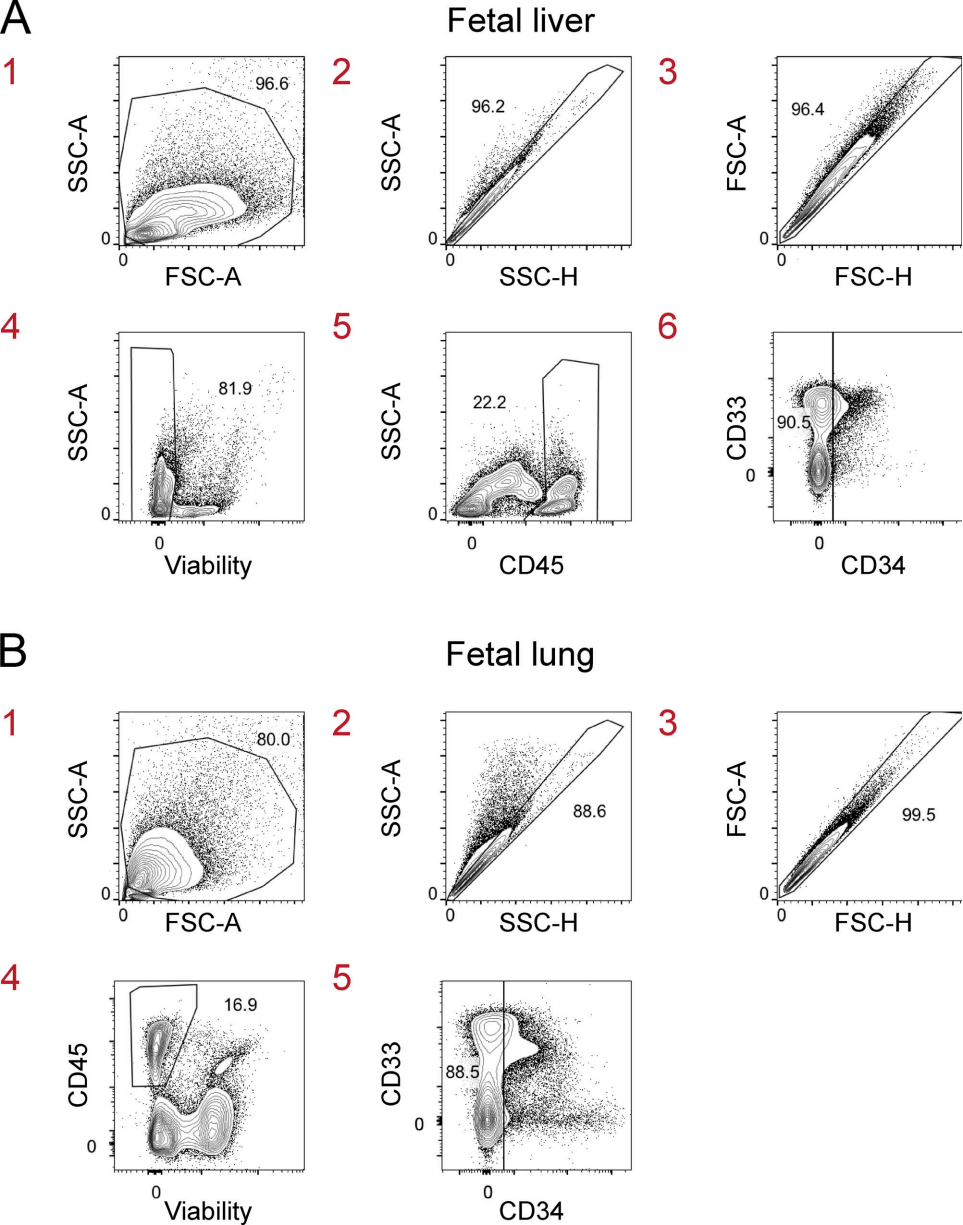
**Gating strategy to identify macrophage precursors in human fetal liver and lung. (A and B)** Single-cell suspensions from human fetal liver (A) and lung (B) at wk 21 and 22 of gestation, respectively, were first gated on live CD45^+^CD34^−^ single cells before gating on macrophage precursors in Fig. 7, A and B. FSC-A, forward scatter area; FSC-H, forward scatter height; SSC-A, side scatter area; SSC-H, side scatter height. Data are representative of two independent experiments. Data (A and B) are representative of two independent experiments with 12 fetal liver and lung samples at 15–23 wk of gestation. The red numbers indicate the order of the flow cytometry plots (sequential gating of cells).

Finally, having identified potential circulating CD116^+^CD64^−^CD14^−^CD115^+^HLA-DR^mid^ alveolar macrophage precursors in MISTRG mice, we hypothesized that corresponding precursors are present in human fetal liver and lung. Consistent with our hypothesis, we found a population of CD116^+^CD64^−^CD14^−^HLA-DR^mid^ cells expressing CD115 as well as the chemokine receptor CX3CR1 in both fetal liver and fetal lung at 15–23 wk of gestation (Fig. 7, A and B; and Fig. S5). These data support the notion that human fetal liver progenitors populate the lung with CD116^+^CD64^−^CD115^+^CX3CR1^+^ alveolar macrophage precursors during the second gestational trimester.

### Human alveolar macrophages derived from fetal and adult precursors have a similar turnover

**Figure 8.**
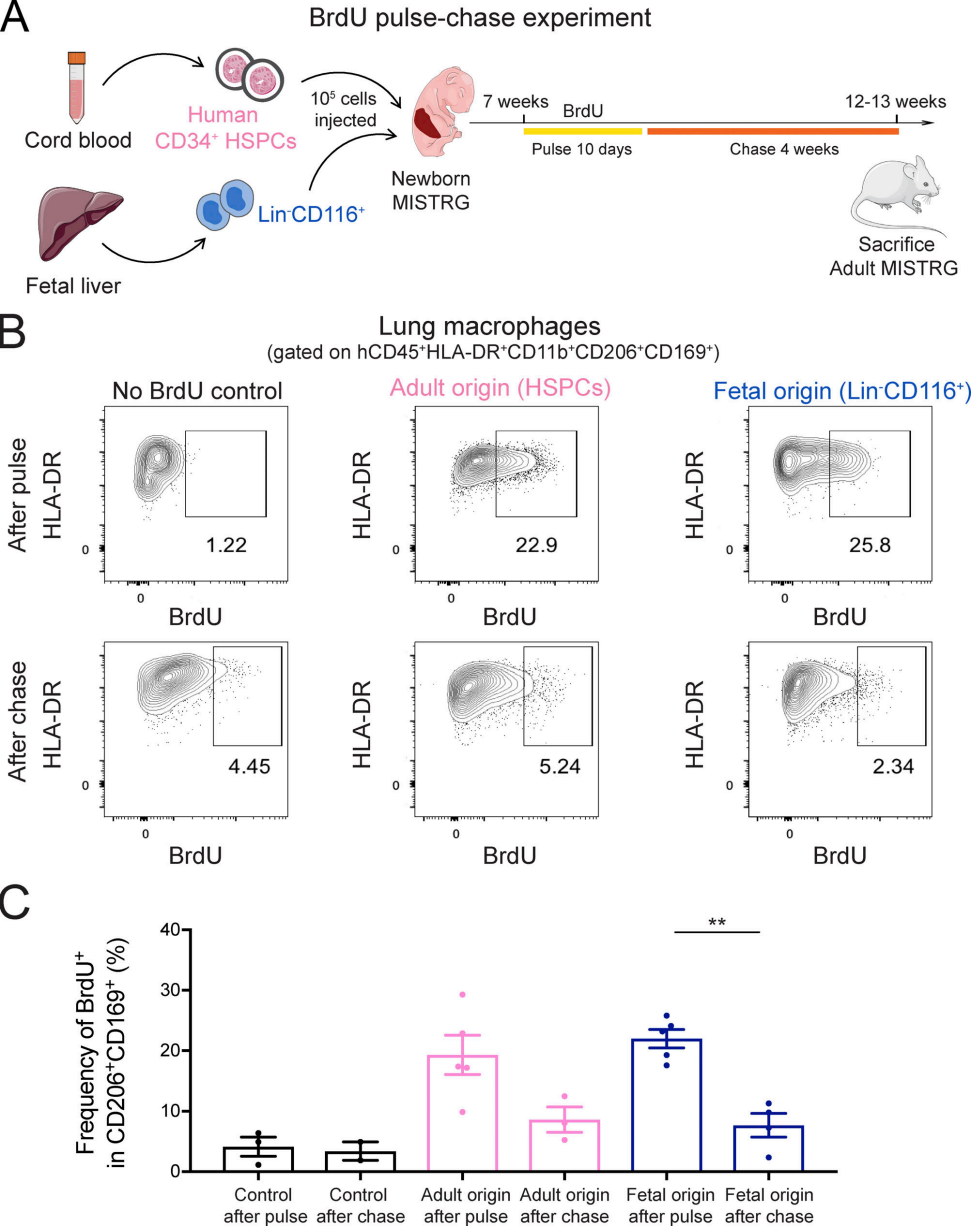
**Human alveolar macrophages derived from fetal and adult precursors have a similar turnover. (A)** Overview of BrdU pulse-chase experiment. MISTRG transplanted with either Lin^−^CD116^+^ fetal liver cells or CD34^+^ HSPCs by intrahepatic injection were pulsed with BrdU for 10 d, followed by a chase period without BrdU for 4 wk. Cartoon was adapted from Servier Medical Art. **(B)** Flow cytometry of BrdU incorporation in human alveolar macrophages of fetal versus adult origin. Numbers indicate the frequency of BrdU^+^ lung macrophages after the pulse and at the end of the chase period. Human lung macrophages were gated as CD45^+^CD11b^+^HLA-DR^+^CD206^+^CD169^+^ cells. Human lung macrophages from mice without BrdU administration were used as a staining control (No BrdU control). **(C)** Frequencies of BrdU^+^ lung macrophages of fetal or adult origin after the pulse and at the end of the chase period. hCD45, human CD45. Data are represented as mean ± SEM. **, P < 0.01 (one-way ANOVA with Tukey’s multiple comparison post hoc test). Data (B) show one lung sample representative of two to five samples (individual mice) per group from two independent experiments. Data (C) are pooled from two independent experiments with *n* = 2–5 per group.

Identifying both the fetal and adult progenitor in this study and in our previous work (Evren et al., 2021) gave us the opportunity to determine the impact of cellular origin on human alveolar macrophage function. Our results above demonstrated that lung macrophages derived from CD34^−^CD116^+^ fetal precursors and from adult precursors (CD34^+^ HSPCs developing into blood monocytes) had a similar capacity to prevent PAP development in MISTRG mice (Fig. 4 F and Fig. 5 E). Next, we compared the turnover of fetal progenitor-derived alveolar macrophages to that of alveolar macrophages of adult origin. For this purpose, we performed pulse-chase experiments with BrdU in MISTRG mice transplanted with either fetal CD34^−^Lin^−^CD116^+^ alveolar macrophage precursors or CD34^+^ HSPCs (Fig. 8 A). BrdU was administered continuously in the drinking water for 10 d to pulse-label alveolar macrophages and BrdU incorporation determined by flow cytometry. We observed a similar labeling frequency (∼20%) of fetal- and adult-derived human alveolar macrophages at the end of the pulse period (Fig. 8, B and C). This showed that a fraction of human alveolar macrophages in MISTRG mice is actively proliferating in situ, similar to what has been reported for mouse alveolar macrophages (Guilliams et al., 2013). After the chase period of 4 wk, the BrdU label was diluted in both fetal- and adult-derived human alveolar macrophages (Fig. 8, B and C), likely through ongoing cell division. These data suggest that, irrespective of their fetal or adult origin, human macrophages reconstituting the alveolar niche in MISTRG mice are maintained by homeostatic proliferation.

### Fetal macrophage precursors outcompete adult monocytes in occupying the perinatal alveolar niche in MISTRG mice

**Figure 9.**
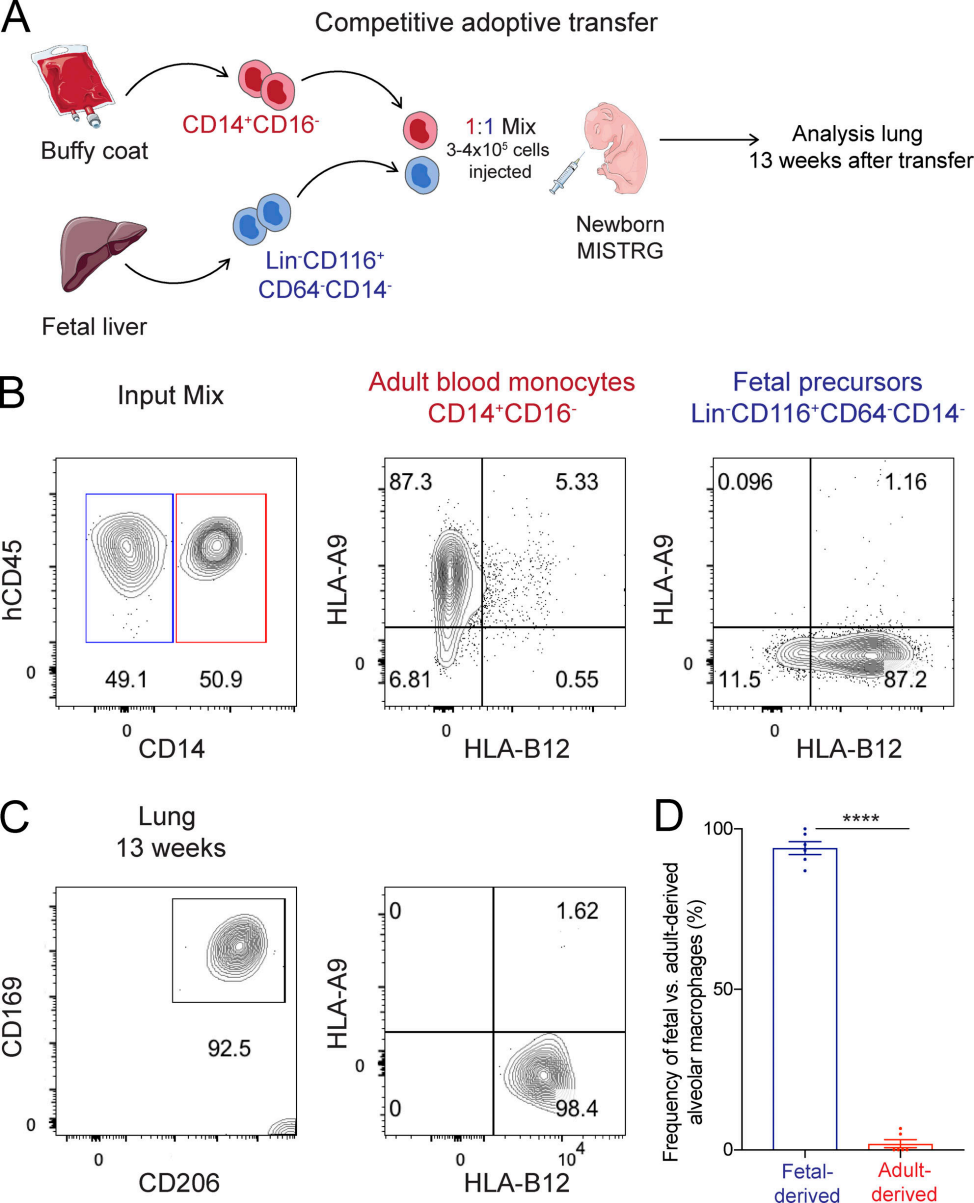
**Fetal macrophage precursors outcompete adult monocytes in occupying the perinatal alveolar niche in MISTRG mice. (A)** Experimental setup of competitive adoptive transfer experiment. Purified fetal and adult macrophage precursors (Lin^−^CD116^+^CD64^−^CD14^−^ fetal liver cells and CD14^+^CD16^−^ blood monocytes) were mixed 1:1 and administered intranasally into newborn MISTRG mice. Cartoon was adapted from Servier Medical Art. **(B)** Flow cytometry of cell mixture before transfer into MISTRG mice to verify the input ratio (left) and to determine surface expression of distinguishing HLA alleles (HLA-A9 versus HLA-B12). **(C)** Flow cytometry of human alveolar macrophages after competitive transfer of fetal and adult macrophage precursors into MISTRG mice. Human lung macrophages were gated as CD45^+^CD11b^+^HLA-DR^+^CD206^+^CD169^+^ cells. Fetal- versus adult-derived lung macrophages were distinguished by surface expression of HLA-A9 and HLA-B12 as in B. **(D)** Frequency of fetal- versus adult-derived human alveolar macrophages in MISTRG mice. hCD45, human CD45. Data are represented as mean ± SEM. ****, P < 0.0001 (unpaired Student’s *t* test). Data (B) show one representative sample from two independent experiments. Data (C) show one lung sample representative of six samples (individual mice) from two independent experiments. Data (D) are pooled from two independent experiments with *n* = 6.

To assess the contribution of precursor origin to the human alveolar macrophage compartment, we performed competitive adoptive transfer experiments with alveolar macrophage precursors (Fig. 9 A). For this purpose, we administered human macrophage precursors into the airways of newborn MISTRG mice, i.e., adult precursors (CD14^+^CD16^−^ monocytes isolated from blood) mixed 1:1 with fetal precursors (CD34^−^Lin^−^CD116^+^CD64^−^CD14^−^ cells purified from fetal liver). We chose the first week of life for these experiments because it is the physiological time when niche colonization by alveolar macrophages occurs in both mice (Guilliams et al., 2013) and humans (Alenghat and Esterly, 1984; Bharat et al., 2016). We then used genetic differences in HLA alleles to distinguish fetal- from adult-derived alveolar macrophages (Fig. 9, B and C). Flow cytometry with HLA allele-specific antibodies demonstrated that fetal precursors outcompeted adult precursors of human alveolar macrophages in terms of their capacity to occupy the alveolar niche in MISTRG mice (Fig. 9, C and D). The preferential expansion of fetal CD116^+^CD64^−^ precursors is consistent with their greater expression of cell cycle genes (Fig. 2, C and D; and Table S1). These results are in line with mouse studies showing that fetal precursors of mouse alveolar macrophages have a greater expansion capacity than adult precursors due to their high intrinsic proliferative potential and metabolic activity (Li et al., 2020; van de Laar et al., 2016). We conclude that human alveolar macrophages of both fetal and adult origin can occupy the alveolar niche in MISTRG mice, have a similar turnover, and are able to clear lung surfactant. However, fetal-derived macrophages have a superior capacity to reconstitute the alveolar compartment in early life.

### Gene signatures of human alveolar macrophages derived from fetal versus adult precursors

**Figure 10.**
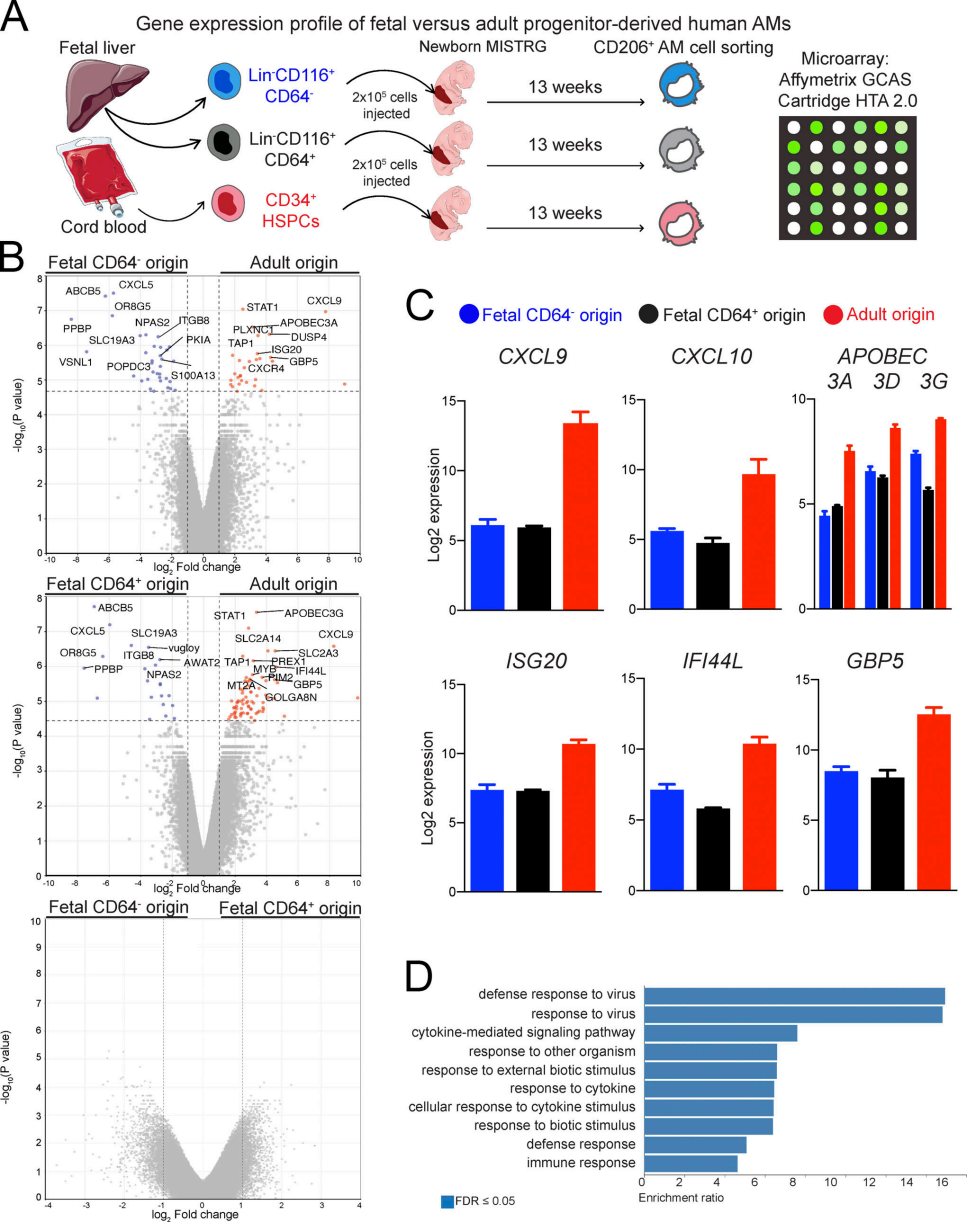
**Gene signatures of human alveolar macrophages of fetal versus origin. (A)** Experimental outline to define the gene expression profiles of human alveolar macrophages (AMs) derived from the indicated fetal and adult precursor populations. Cartoon was adapted from Servier Medical Art. **(B**) Volcano plots of differentially expressed genes (DEGs) between the indicated cell populations. Log2 fold change is plotted versus -log_10_ P value (not corrected for multiple testing). DEGs with a log2 fold change ≥1 and an FDR-corrected P value ≤0.05 are highlighted in blue and red. **(C)** Bar graphs showing expression of selected genes in human lung macrophages derived from fetal precursors (CD116^+^CD64^−^ and CD116^+^CD64^+^ fetal cells) and adult precursors (CD34^+^ HSPCs). Data are represented as mean ± SEM. **(D)** Gene ontology analysis of DEGs up-regulated in human lung macrophages derived from adult precursors compared with fetal precursors.GCAS, GeneChip Array Station; HTA, Human Transcriptome Array. Data (B–D) are from a single microarray experiment with two to three replicates (individual mice) per cell population obtained from three independent cell sorting experiments.

We compared the transcriptomes of fetal- versus adult-derived human lung macrophages to gain insights into potential functional differences. For this purpose, newborn MISTRG mice were transplanted by intrahepatic injection with CD34^+^ HSPCs or with CD34^−^Lin^−^CD116^+^ fetal liver cells that were either CD64^−^ or CD64^+^. 3 mo after transplantation, human alveolar macrophages (CD45^+^CD11b^+^HLA-DR^+^CD206^+^CD169^+^) of different origin were isolated from the lung of MISTRG mice and their gene signatures determined by gene expression profiling (Fig. 10A). The human macrophage populations in MISTRG mice resembled resident macrophages found in the airways of healthy humans (Leach et al., 2020; Morse et al., 2019; Mould et al., 2021; Vieira Braga et al., 2019) as shown by the expression of typical signature genes, such as *MRC1* (encoding CD206), *MARCO*, *FABP4*, *PPARG*, *INHBA*, and *GPNMB* (Table S2). Overall, relatively few genes were differentially expressed between alveolar macrophages of adult origin (HSPC-derived) and alveolar macrophages of fetal origin (derived from either CD64^−^ or CD64^+^CD116^+^ fetal cells; Fig. 10 B and Table S2). This suggests that their gene signatures were largely shaped by cues from the lung environment, consistent with what has been reported for mouse alveolar macrophages (Gibbings et al., 2015; Lavin et al., 2014; van de Laar et al., 2016).

Lung macrophages derived from CD116^+^CD64^−^ and CD116^+^CD64^+^ fetal precursors had very similar transcriptomes with no statistically significant differences in gene expression (Fig. 10 B and Table S2). We therefore focused on comparing human lung macrophages derived from adult precursors with macrophages derived from fetal precursors. Among genes significantly up-regulated in lung macrophages of fetal origin were the chemokines *CXCL5* and *PPBP* (encoding CXCL7; Fig. 10 B and Table S2). Genes with higher expression in lung macrophages of adult origin included the T cell–attracting chemokines *CXCL9* and *CXCL10* as well as IFN-induced genes (*APOBEC3s*, *ISG20*, *IFI44L*, *GBP5*) and the transcription factor *STAT1* (Fig. 10, B and C; and Table S2). This gene signature resembled that of IFN-responsive *CXCL10*-macrophages that we identified previously by single-cell RNA-sequencing in the lung of HSPC-engrafted MISTRG mice (Evren et al., 2021). A similar population of macrophages is associated with lung inflammation and expanded in the airways of humans with severe COVID-19 (Grant et al., 2021; Liao et al., 2020; Mulder et al., 2021; Wauters et al., 2021). Consistent with their host defense function, biological processes such as “defense response to virus” and “response to other organism” were over-represented within the gene signature of adult-derived lung macrophages (Fig. 10 D). We conclude that the local microenvironment has a strong effect on the gene signature of human lung macrophages, while adult precursors preferentially generate pro-inflammatory lung macrophages that have an IFN-induced gene signature.

## Discussion

Alveolar macrophages are essential for lung health, but their ontogeny has been difficult to investigate in humans in view of the inability to perform invasive experiments. We recently demonstrated that CD14^+^ blood monocytes generate human alveolar macrophages in adult life (Evren et al., 2021). In the present study, we defined the development of human lung macrophages from embryonic progenitors and identified CD116^+^ fetal liver cells as precursors of human alveolar macrophages in early life.

Our findings support a model where CD34^−^Lin^−^CD116^+^CD64^−^CD115^+^ macrophage progenitors originating from the fetal liver migrate into the lung, possibly in a CX3CR1-dependent manner, and develop into mature human alveolar macrophages after exposure to tissue-derived GM-CSF and M-CSF. After migration into the lung, CD116^+^CD64^−^ macrophage precursors up-regulated CD64 and other characteristic surface proteins, such as CD206 and CD169, consistent with their differentiation into mature macrophages that clear lung surfactant from the alveoli. We found that human alveolar macrophage precursors in fetal liver already expressed receptors for the key macrophage-instructing cytokines GM-CSF and M-CSF. Moreover, our data indicate that human GM-CSF and M-CSF, produced by the mouse epithelium in MISTRG mice are sufficient to instruct human alveolar macrophage development. TGFβ is another cytokine that promotes the differentiation of mouse alveolar macrophages (Yu et al., 2017), but in contrast with GM-CSF, it does not exclusively derive from a nonhematopoietic source. In MISTRG mice, human TGFβ is likely provided by human alveolar macrophages themselves (Branchett et al., 2021).

Apart from being responsive to instructive cytokines from the local environment, alveolar macrophage precursors need to be able to egress from the fetal liver into the circulation and migrate into the lung. In mice, fetal liver monocytes migrate into tissues in a CCR2-independent manner (Hoeffel et al., 2015; Rantakari et al., 2016), in contrast with adult monocytes (Ly6C^hi^ in mice, CD14^+^ in humans; Guilliams et al., 2018). This underscores that fetal and adult precursors of alveolar macrophages are distinct, in line with our observation that HSPC-derived macrophage precursors, i.e., CD14^+^ blood monocytes (Evren et al., 2021), and CD64^−^CD14^−^ fetal macrophage precursors have different surface phenotypes and gene signatures. Colonization of tissues in the mouse embryo by macrophage precursors requires CX3CR1 and additional, as yet unknown, receptors (Mass et al., 2016). We found CX3CR1 expression on fetal alveolar macrophage precursors, and the CX3CR1 ligand CX3CL1 is expressed in the human fetal and adult lung (Cao et al., 2020; Travaglini et al., 2020). Therefore, CX3CR1 is a potential candidate receptor mediating the trafficking of CD116^+^CD64^−^ macrophage precursors from the fetal liver to the lung.

The surface phenotype of the fetal progenitor of human alveolar macrophages is distinct from known progenitors that give rise to human monocytes during adult hematopoiesis in the bone marrow, such as CD34^+^CD64^mid^ granulocyte-monocyte progenitors (Kawamura et al., 2017), CD34^+^CD115^+^CD116^−^ monocyte-dendritic cell progenitors (Lee et al., 2015), CD34^+^CD64^+^CD115^+^CD116^+^ common monocyte progenitors (Kawamura et al., 2017), and CD34^−^CD64^+^CD115^+^CD116^+^ premonocytes (Breton et al., 2015; Kawamura et al., 2017). Furthermore, CD116^+^CD64^−^CD14^−^ fetal progenitors of human alveolar macrophages are different from CD116^+^CD64^+^ fetal monocytes that express CD14. Instead, the gene signature of fetal CD34^−^Lin^−^CD116^+^CD64^−^ alveolar macrophage precursors suggests that they are the human equivalent of EMPs or downstream MYB^+^CD64^−^ myeloid progenitors, which up-regulate CD64 in response to M-CSF in vitro and give rise to CD64^+^ fetal monocytes and alveolar macrophages in mice (Gomez Perdiguero et al., 2015; Hoeffel et al., 2015; Li et al., 2020; Mass et al., 2016).

We found that both CD116^+^CD64^−^ and CD116^+^CD64^+^ fetal liver cells gave rise to human macrophages with an alveolar macrophage surface phenotype and gene signature. However, only macrophages derived from CD116^+^CD64^−^ fetal precursors efficiently catabolized lung surfactant. Compared with their CD64^+^ counterparts, CD116^+^CD64^−^ fetal liver cells are likely the main progenitor of mature human alveolar macrophages based on their presence in the circulation and ability to migrate from the liver to the lung, their proliferative gene signature associated with efficient occupation of the alveolar niche, and their ability to turn over lung surfactant. It is possible that EMP-like CD116^+^CD64^−^ fetal precursors develop directly into alveolar macrophages. Alternatively, CD116^+^CD64^−^ fetal precursors could further differentiate into CD116^+^CD64^+^ fetal monocytes and then into human alveolar macrophages.

Lung transplantation data suggest that alveolar macrophages in humans are derived from both embryonic precursors and circulating monocytes (Bittmann et al., 2001; Byrne et al., 2020; Eguíluz-Gracia et al., 2016; Kjellström et al., 2000; Nayak et al., 2016). The current work, combined with our previous study (Evren et al., 2021), demonstrates that, in a noncompetitive situation, CD116^+^ fetal liver cells and HSPC-derived CD14^+^ blood monocytes are precursors of human alveolar macrophages in early and adult life, respectively. In mice, fetal monocytes originating from CD115^+^ progenitors in the fetal liver (Hoeffel et al., 2015) seed the lung during the perinatal period and give rise to alveolar macrophages (Guilliams et al., 2013; Schneider et al., 2014; Yu et al., 2017). Similarly, under physiological conditions, CD116^+^CD64^−^ fetal precursors are likely the main progenitor of human alveolar macrophages in early life. Consistent with this idea, we found that fetal macrophage precursors had a greater ability to occupy the alveolar compartment in early life than adult blood monocytes. The observation that human CD116^+^ fetal precursors outcompeted CD14^+^ blood monocytes in populating the perinatal alveolar niche in MISTRG mice is in line with mouse studies (van de Laar et al., 2016) and with the need for fetal macrophage precursors to physiologically occupy the niche shortly after birth in mice (Guilliams et al., 2013) and humans (Alenghat and Esterly, 1984; Bharat et al., 2016) in order to be ready for environmental exposure after birth. Overall, our findings are consistent with the concept that diverse, distinct fetal and adult hematopoietic precursors contribute to tissue-resident macrophages according to timed developmental waves (Ginhoux and Guilliams, 2016).

We not only identified the progenitor of human alveolar macrophages in early life but also addressed the fundamental question of whether cellular origin or cues from the local tissue environment determine the function of lung macrophages, taking advantage of our unique model system to answer this issue in the human context. We found that the local microenvironment shapes the transcriptome of human lung macrophages, irrespective of fetal or adult origin. Similar results have been reported for mouse alveolar macrophages (Gibbings et al., 2015; Lavin et al., 2014; van de Laar et al., 2016), indicating that this is conserved between species. Moreover, our data demonstrate that human macrophages originating from fetal or adult progenitors were both able to reconstitute an empty alveolar niche and to catabolize pulmonary surfactant, similar to what has been reported for mouse alveolar macrophages (van de Laar et al., 2016). Furthermore, mouse studies reported that fetal macrophage progenitors have a greater proliferative capacity than their adult counterparts (Li et al., 2020; van de Laar et al., 2016). Consistent with this concept, we found that fetal CD116^+^CD64^−^ precursors of human alveolar macrophages had higher expression of genes associated with proliferation and outcompeted adult macrophage precursors (CD14^+^ blood monocytes) when reconstituting the alveolar niche. This indicates that fetal-derived human alveolar macrophages may occupy the niche faster than their adult-derived macrophages, but after niche colonization, both populations have a similar turnover and persist long-term.

In the healthy lung, resident macrophages of embryonic origin play an essential anti-inflammatory role by clearing inhaled microbes, dead cells, and surfactant. During severe lung injury, resident macrophages with a tissue-protective function are replaced by macrophages derived from recruited blood monocytes. We found that, compared with their counterparts originating from fetal precursors, lung macrophages derived from adult precursors had a gene signature characteristic of IFN-induced macrophages (Evren et al., 2021) that likely arise from the interaction with T lymphocytes (Mulder et al., 2021). These blood monocyte-derived macrophages are more pro-inflammatory and cause lung damage in important diseases, such as COVID-19 (Bost et al., 2020; Liao et al., 2020; Wauters et al., 2021). The human fetal progenitor that we identified is therefore a potential target to regenerate tissue-protective macrophages in order to limit organ damage and promote tissue repair in the injured lung.

The discovery of human lung macrophage progenitors and their developmental paths is an important step forward. Our in vivo study reveals not only the embryonic origin of human alveolar macrophages but also the impact of cell origin on human lung macrophage specification and function. Therefore, this study provides new insights into the ontogeny of human lung macrophages, which could be used in the long term to develop macrophage-based therapies for important lung diseases in humans.

### Limitations of the study

A limitation of our experimental approach is that cell transplantation into an empty alveolar niche in MISTRG mice mainly assigns progenitor potential. However, the alveolar niche is also physiologically empty during development when macrophage precursors develop into alveolar macrophages early in life. Another limitation is that prenatal events of human lung macrophage development (Miah et al., 2021) cannot be studied and that human macrophage precursors develop in a mouse lung environment with potentially altered species-specific cell–cell interactions. The latter is relevant as environmental cues drive macrophage phenotype and function in different tissues (Amit et al., 2016; Lavin et al., 2014). However, production of human GM-CSF and M-CSF by the mouse lung epithelium is sufficient to support the development of human alveolar macrophages in MISTRG mice. Moreover, certain types of human cells that may regulate alveolar macrophage function, such as basophils (Cohen et al., 2018), might not develop optimally in MISTRG mice. Despite its limitations, our study provides important information about the origin and development of human alveolar macrophages that is difficult to obtain with other approaches.

## Materials and methods

### Mice

MISTRG mice homozygous for the human genes encoding M-CSF, IL-3/GM-CSF, signal regulatory protein α (SIRPα), and thrombopoietin in the *Rag2*^−/−^*Il2rg*^−/−^ 129 × BALB/c (N2) genetic background were previously described (Rongvaux et al., 2014). MISTRG mice were used under Material Transfer Agreements with Regeneron Pharmaceuticals and Yale University. For this study, we used an improved version of MISTRG mice with the *SIRPA* knock-in allele (Deng et al., 2015) instead of the *SIRPA* transgene as in the original MISTRG mice. As recipients for transplantation with human cells (see below), MISTRG mice were used that were either homozygous or heterozygous for *SIRPA* knock-in. Heterozygous mice were derived from breeding MISTRG mice (homozygous for *SIRPA*) with MITRG mice (lacking the *SIRPA* knock-in allele; Rongvaux et al., 2014). MISTRG mice were rederived by embryo transfer at Karolinska Institutet and maintained in individually ventilated cages under specific pathogen–free conditions without any prophylactic antibiotics. Mice (both males and females) were generally used at 7–26 wk after transplantation with human cells. Mice did not receive any irradiation as preconditioning before transplantation. Whenever possible, littermates were used as controls. All mouse experiments were performed in accordance with protocols approved by the Linköping Animal Experimentation Ethics Committee (#29-15, 03127–2020).

### Human tissues

For transplantation of MISTRG mice, frozen fetal liver cells from second trimester were obtained from Yale University collected as part of a previous study (Rongvaux et al., 2014). Umbilical cord blood and buffy coats were obtained from caesarean sections and the Blood Bank at Karolinska University Hospital Huddinge, respectively. The collection of all human tissues was approved by local Ethical Review Boards at Karolinska Institutet (#2006/229-31/3, 2015/1368-31/4, 2015/2122-32, 2016/1415-32, 2018/2162-32) and Yale University (#0804003766). Flow cytometry of fetal liver and fetal lung was performed at Institut Pasteur with fetal tissues (15–23 wk of gestation) that were obtained from Advanced Bioscience Resources Inc. following approval by an institutional medical ethical committee at Institut Pasteur and by the French Ministry of Education and Research (#2018-1003(10)M1). Informed consent was obtained from all tissue donors following verbal and written information, and the investigations were conducted according to the Declaration of Helsinki.

### Transplantation of MISTRG mice with human cells

For transplantation with human HSPCs, CD34^+^ cells were isolated from pooled cord blood by density gradient centrifugation and positive immunomagnetic selection using a CD34^+^ microbead kit (Miltenyi Biotec). Newborn MISTRG mice (3–5 d old) were transplanted with 10^5^ human CD34^+^ cells (usually >90% purity) by intrahepatic injection as previously described (Evren et al., 2021). HSPCs were pooled from several donors for transplantation.

For transplantation with human CD34^−^ fetal liver cells, the CD34^−^ fraction of fetal liver was obtained after isolation of CD34^+^ cells by density gradient centrifugation and positive immunomagnetic selection using a CD34^+^ microbead kit (Miltenyi Biotec) as described (Rongvaux et al., 2014). Frozen CD34-depleted fetal liver cells were then further purified by cell sorting into the indicated cell populations (purity ≥95%). Newborn MISTRG mice (3–5 d old) received purified cells either via the intranasal route (0.5–1 × 10^5^ cells in 7 µl PBS) or by intrahepatic injection (10^5^ cells in 20 µl PBS). Control mice received PBS only or no cell injection as indicated. *SIRPA* homozygous and heterozygous MISTRG mice were used for intranasal and intrahepatic cell transfer, respectively. Recipient mice were analyzed at the indicated times after cell transfer.

For competitive precursor transfer experiments, CD14^+^CD16^−^ blood monocytes were purified from buffy coats as described (Evren et al., 2021). Blood monocytes were first enriched by negative immunomagnetic selection using EasySep Human Monocyte Enrichment kit (StemCell Technologies, Inc.), and then CD14^+^CD16^−^ monocytes were further purified by cell sorting (purity ≥95%). CD45^+^CD34^−^Lin^−^CD116^+^CD64^−^CD14^−^ cells were purified by cell sorting from the CD34^−^ fraction of fetal liver (purity ≥95%). Purified macrophage precursors (CD14^+^CD16^−^ blood monocytes and CD116^+^CD64^−^ fetal liver cells) were mixed 1:1 for adoptive transfer and administered intranasally into newborn MISTRG mice (3–4 × 10^5^ cells in total). The input ratio was confirmed by flow cytometry based on CD14 surface expression. Surface expression of HLA-A and HLA-B alleles was determined for each cell origin (buffy coat or fetal liver) by flow cytometry with a panel of allele-specific antibodies. Cell origin of human lung macrophages obtained from MISTRG mice was determined with allele-specific HLA-A and HLA-B antibodies 13 wk after transfer.

### Isolation of immune cells from transplanted MISTRG mice

Lungs were perfused with 10 ml ice-cold PBS and digested in RPMI 1640/5% FCS with 0.2 mg/ml of collagenase IV (Sigma-Aldrich) and 0.02 mg/ml of DNase I (Sigma-Aldrich) for 60 min at 37°C. Digested cells were then passed sequentially through 18- and 20-G needles before density gradient centrifugation using Lymphoprep (StemCell Technologies, Inc.). BAL fluid was collected by inflating the lungs three times with 0.8 ml PBS via a catheter inserted into the trachea. BAL fluid was then centrifuged, the pellet resuspended in RPMI 1640/5% FCS, and BAL cells purified for flow cytometry by density gradient centrifugation. The supernatants were frozen at −80°C and the total protein concentration in BAL supernatants determined using the Bicinchoninic acid assay kit (Thermo Fisher Scientific) according to the manufacturer’s instructions. Blood was taken by cardiac puncture and diluted in 200 U/ml heparin (Sigma-Aldrich). Erythrocytes were removed using red blood cell lysis buffer (obtained from Karolinska University Hospital) and the remaining immune cells stained for flow cytometry analysis.

### Cell isolation from fetal liver and fetal lung for flow cytometry

Fetal lungs were cut into small pieces and digested in RPMI 1640 (Life Technologies) with 25 µg/ml Liberase TL (Roche), 100 µg/ml DNase I (Roche), 100 U/ml penicillin, and 100 µg/ml streptomycin (Thermo Fisher Scientific) for 60 min at 37°C and filtered through a 100-µm cell strainer (Corning). Fetal livers were cleaned from vascular tissue, paddle-blended in a sterile bag, filtered through a 100-µm cell strainer (Corning), and centrifuged on a Ficoll (GE Healthcare) density gradient. Mononuclear cells were recovered and CD34^+^ cells depleted using the CD34^+^ microbead kit (Miltenyi Biotec). Cell preparation steps were performed using RPMI 1640 supplemented with 100 U/ml penicillin, 100 µg/ml streptomycin, and 2% FCS unless indicated otherwise. Cells were frozen in FCS with 10% DMSO (Sigma-Aldrich) at −80° until used for flow cytometry.

### Flow cytometry and cell sorting

Single-cell suspensions from human fetal liver and lung as well as from lung and blood of MISTRG mice were stained with fluorochrome- or biotin-labeled anti-human antibodies in FACS buffer (PBS/2% FCS) for 30 min on ice, followed by secondary staining with streptavidin–Brilliant Violet 711 (BD Biosciences) for 30 min on ice. To exclude T cells, B cells, natural killer cells, and granulocytes, a Lin cocktail was used, which consisted of CD3, TCRαβ, TCRγδ, CD19, CD20, CD56, CD94, NKp46, and CD66abce (for flow cytometry of fetal liver and lung) or CD3, CD19, CD56, NKp46, and CD66abce (for cell sorting and flow cytometry of MISTRG mice). For staining of fetal liver and fetal lung, Fc receptors were blocked using IgG from human serum (Millipore Sigma) and nonspecific dye binding inhibited by using True-Stain Monocyte Blocker solution (BioLegend). After surface staining, cells were stained with fixable viability dye eFluor506 or eFluor455UV (Thermo Fisher Scientific) according to the manufacturer’s instructions. Cells were fixed in PBS/2% paraformaldehyde and acquired on a LSR II Fortessa or Symphony A5 flow cytometer (BD Biosciences), and data were analyzed with FlowJoV10 software. The indicated cell populations were sorted into RPMI 1640/30% FCS medium using a BD FACSAria III (BD Biosciences) or a MA900 (Sony Biotechnology) cell sorter.

The following antibodies were used for flow cytometry and cell sorting: biotinylated anti-human CD3 (OKT3; Thermo Fisher Scientific or UCHT1; BioLegend), CD19 (HIB19; Thermo Fisher Scientific or BioLegend), CD20 (2H7; BioLegend), CD56 (HCD56; BioLegend), CD66abce (REA1230 or TET2; Miltenyi Biotec), CD94 (REA113; Miltenyi Biotec), NKp46 (9E2; BioLegend), TCRαβ (IP26; BioLegend), TCRγδ (B1; BD Biosciences), HLA-B12 (REA138; Miltenyi Biotec); conjugated anti-human CD1c BV785 (L161; BioLegend), CD1c BV650 (L161; BioLegend), CD11b PerCP-Cy5.5 (ICRF44; BioLegend), CD11b BUV737 (M1/70; BD Biosciences), CD11b BB515 (ICRF44; BD Biosciences), CD11c R718 (B-ly6; BD Biosciences), CD14 BV421 (M5E2; BioLegend), CD14 BV480 (MϕP9; BD Biosciences), CD16 PE-Cy7 (3G8; BioLegend), CD33 BUV805 (WM53; BD Biosciences), CD34 PE-Dazzle594 (581; BioLegend), CD34 BV650 (563; BD Biosciences), CD34 FITC (581; BD Biosciences), CD45 APC-Cy7 (HI30; BioLegend), CD45 BUV395 (HI30; BD Biosciences), CD45 BV785 (HI30; BioLegend), CD64 BUV737 (10.1; BD Biosciences), CD64 PE-Dazzle594 (10.1; BioLegend), CD88 APC (S5/1; BioLegend), CD88 PE-Cy7 (S5/1; BioLegend), CD115 PE-Cy7 (9-4D2-1E4; BioLegend), CD116 PE (4H1; Thermo Fisher Scientific or BioLegend), CD141 BV785 (M80; BioLegend), CD141 BV650 (1A4; BD Biosciences), CD163 BV650 (GHI/61; BD Biosciences), CD169 APC (7–239; BioLegend), CD169 BV421 (7–239; BioLegend), CD206 BV605 (19.2; BD Biosciences), CX3CR1 BB515 (2A9-1; BD Biosciences), HLA-A2 AF700 (BB7.2; BioLegend), HLA-A9 APC (REA127; Miltenyi Biotec), HLA-B8 APC-Cy7 (REA145; Miltenyi Biotec), HLA-DR BUV395 (G46-6; BD Biosciences), HLA-DR APC-Fire750 (L243; BioLegend), HLA-DR BV650 (L243; BioLegend), and conjugated anti-mouse CD45 AF700 (30-F11; BioLegend).

### Immunohistochemistry

PBS-perfused lungs were collected, fixed in 4% paraformaldehyde for 24–48 h, and then stored in 70% ethanol. Dehydrated lungs were embedded in paraffin and cut into 5-µm sections. Lung sections were rehydrated by successive washes in xylene (Sigma-Aldrich), 100% ethanol, 95% ethanol, 70% ethanol, and 50% ethanol. Heat-induced epitope retrieval was performed in citrate buffer (10 mM citrate, pH 6.0), and excess aldehyde was quenched with 0.2 M glycine. After blocking with 10% normal goat serum (Dako), sections were stained with mouse anti-human CD68 antibody (clone PG-M1; Dako), diluted 1:100 in 2% normal goat serum/PBS overnight at 4°C, followed by a biotinylated goat anti-mouse IgG secondary antibody (Dako), diluted 1:200 in 2% normal goat serum/PBS for 1 h at room temperature. Endogenous peroxidase activity was removed by an additional blocking step in 1% hydrogen peroxide/methanol (Sigma-Aldrich). Staining was revealed with the DAB Peroxidase (HRP) Substrate Kit and Vectastain Elite Kit (both from Vector Labs). Finally, slides were counterstained in hematoxylin (Sigma-Aldrich), dehydrated, and mounted using Permount.

### Macrophage turnover

Newborn MISTRG pups were transplanted intrahepatically with either 10^5^ CD34^+^ HSPCs or Lin^−^CD116^+^ fetal liver cells and then used for BrdU pulse-chase experiments 7 wk after transplantation. For the BrdU pulse, transplanted MISTRG mice received an initial intraperitoneal injection of 1.5 mg BrdU (BD Biosciences) per mouse, followed by continuous BrdU treatment (0.8 mg/ml in drinking water supplemented with 10% sucrose) for 10 d (with fresh BrdU water prepared every 1–2 d). Mice were then either sacrificed following the pulse period or kept on drinking water without BrdU for a chase period of 4 wk. BrdU incorporation was determined in human CD11b^+^HLA-DR^+^CD206^+^CD169^+^ lung macrophages after pulse and after chase by flow cytometry using the BrdU APC Flow Kit (BD Biosciences). Human lung macrophages from HSPC-engrafted MISTRG mice that were not treated with BrdU were used as staining control to set up the BrdU^+^ gate.

### Gene signatures of human lung macrophages and their precursors

To define the gene signature of adult precursors of human alveolar macrophages, CD14^+^CD16^−^ blood monocytes were purified from buffy coats by immunomagnetic selection and by cell sorting as described above. To define the gene signatures of fetal precursors of human alveolar macrophages, fetal monocytes (CD45^+^CD34^−^Lin^−^CD116^+^CD64^+^CD88^+^CD1c/CD141^lo^CD206^−^CD169^−^) and fetal precursor-like cells (CD45^+^CD34^−^Lin^−^CD116^+^CD64^−^CD1c/CD141^−^CD14^−^) were purified from the CD34^−^ fraction of fetal liver by cell sorting. Both adult and fetal precursors (purity ≥95%) were directly sorted into RLT buffer or RLT plus buffer (Qiagen). Adult blood monocytes and fetal alveolar macrophage precursors had each three replicates, while there were four replicates for fetal monocytes, of which two replicates were pooled together after RNA extraction for a total of three replicates.

To define the gene signatures of human macrophages of different precursor origin, human cells with an alveolar macrophage surface phenotype (CD45^+^CD11b^+^HLA-DR^+^CD206^+^CD169^+^) were purified from MISTRG mice 13 wk after intrahepatic transplantation of newborn mice with either adult (2 × 10^5^ CD34^+^ HSPCs; three replicates) or fetal precursors (2 × 10^5^ CD34^−^Lin^−^CD116^+^CD64^−^ or CD34^−^Lin^−^CD116^+^CD64^+^ fetal liver cells; three and two replicates, respectively). HSPC-engrafted MISTRG mice were injected i.v. with 2 µg of PE-conjugated anti-human CD45 antibody (HI30; BioLegend) before lung harvest to exclude i.v. CD45-PE^+^ pulmonary intravascular macrophages from the sorted CD45^+^CD11b^+^HLA-DR^+^CD206^+^CD169^+^ subset as described (Evren et al., 2021).

Samples were sent to the Bioinformatics and Expression Analysis Core Facility at Karolinska Institutet for RNA extraction, amplification, and hybridization to human Clariom D cartridge microarrays (Applied Biosystems). Transcriptome Analysis Console software (Thermo Fisher Scientific) was used for the analysis of the microarray data. Signal values were log2-transformed and normalized using the Signal Space Transformation–Robust Multiple-array Average method. Gene expression between different cell populations was compared using ANOVA with eBayes analysis. Multiple testing correction was performed based on Benjamini–Hochberg to calculate false discovery rate (FDR)–corrected P values. Genes with a fold change ≥2 and an FDR-corrected P value <0.05 were considered differentially expressed between paired cell populations. Gene Ontology over-representation analysis (Biological Process) was performed with WebGestalt (http://webgestalt.org/) using default parameters.

The gene expression data are available in GEO under accession nos. GSE190256 (fetal and adult precursors of human alveolar macrophages) and GSE190257 (human alveolar macrophages of fetal versus adult origin).

### Bead-based fate-mapping of circulating lung macrophage precursors

To fate-map circulating macrophage precursors, MISTRG mice were injected i.v. as described (Evren et al., 2021) with 100 µl of 0.5-µm-microsphere Fluoresbrite PC red/PE beads (Polysciences; diluted 1:10) 5–7 wk after intrahepatic transplantation with Lin^−^CD116^+^ fetal liver cells. With this protocol, injected beads were captured by CD116^+^CD64^−^ circulating precursors, the cell subset present predominantly in the blood at this time point. Mice were sacrificed 7 d after bead injection, i.e., 6–8 wk after initial cell transplantation, and lungs harvested to determine the frequency of fluorescent bead^+^ lung macrophages by flow cytometry. Human lung macrophages from HSPC-engrafted MISTRG mice that were not injected with beads were used as staining control for the bead^+^ gate.

### Quantification and statistical analysis

Statistical parameters including number of biological replicates and repeat experiments, data dispersion and precision measures (mean and SEM), and P values for statistical significance (α = 0.05) are reported in the figures and figure legends. Student’s *t* test was used to determine statistical significance between two groups. For multigroup comparisons, we applied one-way ANOVA with post hoc testing using Tukey’s multiple comparison test. Statistical analysis was performed using GraphPad Prism 7.

### Online supplemental material

Fig. S1 shows the gating strategy for flow cytometry of fetal liver cells in Fig. 1. Fig. S2 shows the flow cytometry gating strategy for human hematopoietic cells in the lung and blood of MISTRG mice in Figs. 3, 4, 5, and 6. Fig. S3 shows the immunohistochemistry controls for Figs. 3 and 4. Fig. S4 shows flow cytometry for human lung macrophages with an interstitial surface phenotype in MISTRG mice transplanted with either CD116^+^ fetal liver cells or HSPCs. Fig. S5 shows the flow cytometry gating strategy for fetal lung and fetal liver cells in Fig. 7. Table S1 (related to Fig. 2) contains the gene expression profiles of CD116^+^CD64^−^ fetal precursors and CD116^+^CD64^+^ fetal monocytes from human fetal liver as well as the gene expression profile of CD14^+^CD16^−^ adult monocytes from human blood. Table S2 (related to Fig. 10) contains the gene expression profiles of human lung macrophages derived from fetal precursors (CD116^+^CD64^−^ or CD116^+^CD64^+^ fetal liver cells) or from adult precursors (CD34^+^ HSPCs) in MISTRG mice.

## Supplementary Material

Table S1 shows gene expression profiles of human CD14^+^ blood monocytes, CD116^+^CD64^+^ fetal liver monocytes, and CD116^+^CD64^−^ fetal alveolar macrophage (AM) precursors.

Table S2 shows gene expression profiles of human lung macrophages derived from CD116^+^CD64^+^ fetal monocytes, CD116^+^CD64^−^ fetal precursors, and CD34^+^ HSPCs in MISTRG mice.
